# *Moringa oleifera* leaf supplementation relieves oxidative stress and regulates intestinal flora to ameliorate polycystic ovary syndrome in letrozole‐induced rats

**DOI:** 10.1002/fsn3.3473

**Published:** 2023-06-06

**Authors:** YanXiang Wu, XiuYan Yang, YuanYuan Hu, XueHong Hu, YueLin Zhang, Tian An, BoHan Lv, SiYu Tao, Qing Liu, GuangJian Jiang

**Affiliations:** ^1^ Traditional Chinese Medicine School Beijing University of Chinese Medicine Beijing China; ^2^ School of traditional Chinese medicine Capital Medical University Beijing China; ^3^ Beijing Changping Qingyitang Hospital of Traditional Chinese Medicine Beijing China; ^4^ Beijing Yaoshi Tongyuan Trading Co., Ltd. Beijing China

**Keywords:** inflammation, intestinal flora, *Moringa oleifera* leaf, oxidative stress, polycystic ovary syndrome

## Abstract

This study investigated the effects of supplementation *Moringa oleifera* leaf (MOL) on relieving oxidative stress, anti‐inflammation, changed the relative abundance of multiple intestinal flora and blood biochemical indices during letrozole‐induced polycystic ovary syndrome (PCOS). Previous studies have shown that MOL has anti‐inflammatory, anti‐oxidation, insulin‐sensitizing effects. However, whether MOL has beneficial effects on PCOS remains to be elucidated. In the current study, 10‐week‐old female Sprague–Dawley rats received letrozole to induce PCOS‐like rats, and subsequently were treated with a MOL diet. Then, the body weight and estrus cycles were measured regularly in this period. Finally, the ovarian morphology, blood biochemical indices, anti‐oxidative, intestinal flora, and anti‐inflammation were observed at the end of the experiment. We found that MOL supplementation markedly decreased the body weight, significantly upregulated the expression of Sirt1, FoxO1, PGC‐1α, IGF1, and substantially modulated the sex hormone level and improved insulin resistance, which may be associated with the relieves oxidative stress. Moreover, the supplementation of MOL changed the relative abundance of multiple intestinal flora, the relative abundance of *Fusobacterium*, *Prevotella* were decreased, and *Blautia* and *Parabacteroides* were increased. These results indicate that MOL is potentially a supplementary medication for the management of PCOS.

## Graphical abstract

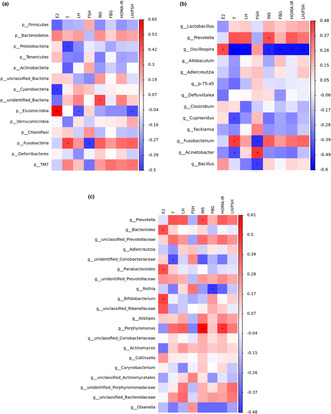

## 1 BACKGROUND

Polycystic ovary syndrome (PCOS) is a reproductive endocrine disorder, which is associated with an increased risk of many chronic disease (Meyer et al., 2012; Teede et al., 2015). Although the etiology and pathogenesis of PCOS are still not fully completely elucidated, hyperandrogenism, insulin resistance (IR), obesity, dysregulation of glucose metabolism, inflammation, and oxidative damage play a central role (Ostadmohammadi et al., 2019). Reactive oxygen species (ROS) play an important role in cellular functions under both physiological and pathological conditions. Oxidative stress is an imbalance between ROS and antioxidant defense systems in the cell, which leads to inflammatory infiltration of neutrophils and increased secretion of proteases (Sanford et al., 2020). Studies reported that oxidative stress and inflammation may be the pathogenesis mechanisms affecting PCOS (Sabuncu et al., 2001). Oxidative stress not only can affect the normal structure and function of follicles but also inhibit follicular development, maturation, and expulsion occurs of oocytes in PCOS patients (Snider & Wood, 2019). As revealed by a recent study PCOS was accompanied by oxidative stress that may result from obesity, hyperinsulinemia, and dyslipidemia. The important contribution of the gut microbiota to human health and disease is widely recognized (Aron‐Wisnewsky & Clément, 2016). Guo et al. revealed that PCOS is closely related to intestinal flora (Guo et al., 2016). In addition, there is evidence that oxidative stress affected the gut microbial composition and metabolism and consequently the gut environment (Liu, Wu, et al., 2019). Therefore, it may be possible to inhibit oxidative stress by improving the dysbiosis, or compositional changes in the intestinal microbiota.

Herbal plants and their ingredients and different balance nutrients have been explored to reduce obesity and have high antioxidant properties, which may be used as potential food supplements for patients with PCOS. *Moringa oleifera* (MO) is endemic in the tropics with a multi‐purpose herbal plant and ethnomedicinal importance and a variety of pharmacological activities for numerous health benefits. *Moringa oleifera* leaf (MOL) are the most nutritious part which possess several biological actions, not only anti‐inflammatory, anti‐microbial, and antioxidant, but also anti‐obesity and anti‐diabetes (Afzal et al., 2021; Yasoob et al., 2021). MOL acts by increasing the oxidative metabolism through the SIRT1‐PPARα pathway. SIRT1 is involved in the activation related to redox homeostasis of transcription factors (Ceci et al., 2022; Duranti, Maldini, Crognale, et al., 2021; Duranti, Maldini, Crognale, Sabatini, et al., 2021). Previous studies have proved that MOL includes diverse bioactive compounds and an abundance of amino acids, like the flavonoid (myricetin, quercetin, and kaempferol) and phenolic acid (chlorogenic acid) (González‐Burgos et al., 2021; Vergara‐Jimenez et al., 2017). Albrahim et al. indicated that MOL extract can exert antioxidant and anti‐inflammatory effects that could induce cellular protection (Albrahim & Binobead, 2018). The therapeutic effect of MOL on diabetes has been evaluated because it contains polyphenols, such as quercetin‐3‐glycosides, rutin, kaempferol, and glycosides, which can reduce blood sugar levels after ingestion. However, studies on MOL for PCOS have been rarely reported. Regarding this, the objective of this study was designed to probe the influence of MOL on relieving oxidative stress, anti‐inflammatory, change the intestinal flora and blood biochemical indices during PCOS of letrozole‐induced rats.

## 2 MATERIALS AND METHODS

### 2.1 Materials

*Moringa oleifera* leaf was purchased from commercial sources (Xishuangbanna, Yunnan Province, China). The food of MOL was prepared at the Diabetes Research Center of Beijing University of Chinese Medicine. *Moringa* powder was sufficiently mixed with water to prepare standard food with shapes and sizes. Putting in the oven, baking at 250 w for 4 min, then taking it out to air dry and storing at 4°C. Letrozole (LET) was purchased from Jiangsu Hengrui Medicine Co., Ltd., Jiangsu (gyzz H199991001, China), Daine‐35 was purchased from Bayer Weimar GmbH und Co. KG (gyzz j20140114, Germany), Anti‐Sirt1 (13161‐1‐AP, US), anti‐FoxO1 (18592‐1‐AP, US), anti‐PGC1α (66369‐1‐Ig, US), anti‐IGF1 (A11985, China), anti‐TLR4 (A5258, China), goat anti‐rabbit IgG H&L (HRP) (AS014, China), and goat anti‐mouse IgG H&L (HRP) (GB23301, China). The DAB kit (ZLI‐9018, China), bicinchoninic acid (BCA) kit, and enhanced chemiluminescence (ECL) kit were purchased from Pierce Biotechnology (IL, USA).

### 2.2 MII oocyte complete transcriptome sequencing

This study was approved by The Chinese Ethical Science Committee (Maternity and Child Care Hospital of North China University of Science and Technology) and conducted in accordance with the Helsinki Declaration and all participants gave informed consent before inclusion in the study. Two oocytes of PCOS patient and two oocytes of normal woman were included. Exclusion criteria were diabetes type 1 or 2, impaired thyroid, renal or hepatic function, congenital adrenal hyperplasia endometriosis, premature ovarian failure, hypothalamic amenorrhea, or age > 35 years. Diagnosis of PCOS was made according to the Rotterdam Consensus Criteria (Snider & Wood, 2019). MII oocytes were collected separately from the patient with PCOS and the normal woman. Oocytes were frozen in liquid nitrogen Jar. The MII oocytes were used for whole‐transcriptome sequencing.

### 2.3 Network pharmacology

Components of MOL were screened by searching PubMed and China National Knowledge Infrastructure (CNKI) database. The target genes of active components of MOL were obtained by the SwissTargetPrediction (http://www.swisstargetprediction.ch) and the candidate targets of PCOS were obtained from Genecards Databases (http://www.genecards.org/). The screened chemical targets and disease targets database were imported into the Cytoscape 3.9.1 platform (were imported into Cytoscape (44) (version 3.3.1)) for analysis, and the common targets of compound‐disease were obtained as the potential targets for further analysis. Venny 2.1.0 (https://bioinfogp.cnb.csic.es/tools/venny) was used to take the intersection of the targets of MOL and PCOS and import them into STRING database 11.5 (https://cn.string‐db.org/)to obtain the PPI network information, and then visualize them to obtain the core target. The common targets were imported into the Metascape database (http://metascape.org/gp/#/main/step1) for GO and KEGG enrichment analysis, and the data were visualized. Using the information on the top ten pathways of KEGG, the component‐target‐pathway map was made to obtain the core components.

### 2.4 Molecular docking

The 3D structure information of the components of MOL was obtained through the PubChem database. At the same time, the 3D structure of the target protein was downloaded through the PDB database (https://www.rcsb.org/). The small molecule and the target protein were pretreated with the Autodocktools 1.5.7 software respectively, such as hydrogenation and decharging. The docking active site was found with the POCASA 1.1 software plug‐in, and the processed component and the target protein could be molecular docked with the Autodock Vina 1.2.0 software, Finally, the docking results were visualized by PyMOL 2.5.2 software.

### 2.5 Animals and treatments

All animal experimental procedures were approved by the Committee for Laboratory Animal Care and Use of Beijing University of Chinese Medicine and conducted in strict compliance with the National Institutes of Health Guide for the Care and Use of Laboratory Animals. Ten‐week‐old female Sprague–Dawley (SD) rats were obtained from Beijing Vital River Laboratory Animal Technology Co., Ltd. (Beijing, China). Rats were maintained in a controlled environment (22–25 °C, 12 h light;12 h dark cycle) under specific pathogen‐free conditions. Meanwhile, standard laboratory rodent food and water were available ad libitum during the phase of the study. After 7 days, 22 rats were randomly divided into four groups: Normal group (C, *n* = 6), PCOS group: LET (L, *n* = 6), Positive group: LET+Diane‐35 (P, *n* = 5), treatment group: LET+ MOL (MOL, *n* = 6). As shown in Figure 9a, the L, P, and MOL group rats were administered orally with LET (1 mg/kg/day) for 25 days, the P group was orally administered with Daine‐35 (0.2 mg/kg/day), and the MOL group was supplementation MOL (500 mg /kg/day) during the evening for 7 weeks. The body weight was measured every week. All rats were euthanized upon the termination of the experiment. Then serum samples were isolated by centrifugation at 3000*g* for 10 min and were stored at −80°C, and the bilateral ovaries, and colon were collected for further analysis.

**FIGURE 1:**
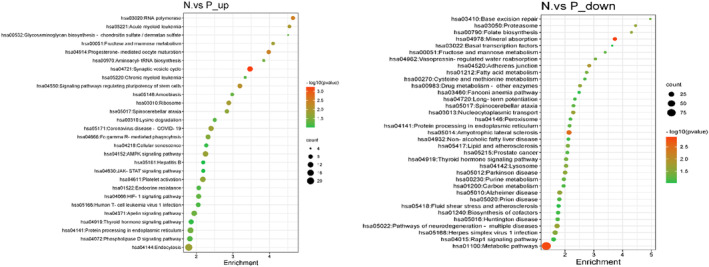
Enrichment analysis for KEGG enrichment analysis of differential genes.

### 2.6 Vaginal smear

Determination of the estrous stage in females was performed by assessing vaginal smear cytologies (Albrahim & Binobead, 2018; Caligioni, 2009). Briefly, the vaginal smear samples were collected between 8:30 and 10:30 am. The tip of a moistened cotton swab was gently inserted into the exterior portion of the vaginal canal (about 1.2 cm–1.5 cm deep) and then rotated to dislodge cells from the vaginal wall, and transferred to glass slides to air dry. Samples were collected over 2–3 consecutive cycles to ensure observation of multiple estrus cycle stages. The cycle stage was determined by visual inspection of cells under a bright‐field microscope (Olympus BX53).

### 2.7 Blood metabolite profile analysis

Blood biochemical tests include estradiol (E2), luteinizing hormone (LH), follicle‐stimulating hormone (FSH), testosterone (T), and the fasting insulin index (FINS). All serum levels were measured by radioimmunoassay (RIA) using RIA kits (Mibio), and all biochemical assays were performed according to the manufacturer's instructions. Fasting blood glucose (FBG) was measured by the Hitachi‐7600 automatic biochemical analyzer. Repeatability analysis was also performed for each sample. The homeostasis model of assessment for IR (HOMA‐IR) was calculated according to the following formula: [FINS (μIU/mL) × FBG (mmol/L)/22.5].

### 2.8 Histological examination and immunohistochemistry (histology)

Ovaries and colons were post fixed at 4% paraformaldehyde overnight, then gradient alcohol dehydration and embedded in paraffin. The 3‐μm thick ovarian and colon tissue sections were deparaffinized, rehydrated, deparaffinized and stained with hematoxylin and eosin was applied for histopathological examination. The images were acquired using an Olympus BX53 microscope (Japan).

Immunohistochemistry was performed to detect the expression of Sirt1, FoxO1, TLR4, and IGF1 in ovary tissue and the claudin‐1, occludin, and TLR4 in colon tissue. Briefly, the section was permeabilized with 1% Triton X‐100 in phosphate buffered saline (PBS) for 30 min at room temperature, boiled in 100 mM sodium citrate (pH 6.0) for 8 min for antigen retrieval, washed in three times with PBS (pH 7.4) and incubated with 0.3% H_2_O_2_ in methanol for 15 min, 10% goat serum incubated 30 min, and Stir1, FoxO1, TLR4, IGF1, claudin‐1 and occludin antibody at 4°C overnight. Subsequently, washing three times in PBS, sections were incubated with IgG H&L (HRP) antibody conjugate for 40 min before chromogenic detection using DAB kit.

### 2.9 Western blotting

Protein expression was determined by western blotting. The ovaries of rat were collected, sample preparation (protein extraction and measurement of protein concentration) from ovaries tissue lysates. The total protein concentration was determined using BCA kits, 20 μg of protein was used for western blot analysis. Denatured proteins were fractionated by SDS–polyacrylamide gel electrophoresis and transferred to a polyvinylidene difluoride (PVDF) membrane. The membranes were blocked with 5% (w/v) non‐fat milk powder in Tris‐buffered saline containing 0.1% (v/v) Tween‐20 (TBST) for 60–90 min and Primary antibody (anti‐Stir1, anti‐FoxO1, anti‐PGC‐1α, and anti‐TLR4) was incubated overnight at 4°C. They were washed three times (5 min each) with TBST, and the membranes were incubated with secondary antibody conjugated with labeled chemiluminescent (anti‐mouse IgG H&L (HRP) or anti‐rabbit IgG H&L (HRP)) for 1 h. The bands were visualized using an ECL kit and detected using an Azure c500 Imaging System (BIO‐RAD) and quantified by molecular imager Image Lab software (ImageJ).

### 2.10 Microbiome analysis

Genomic DNA from rat feces samples was extracted using a specific DNA extraction kit, and then the DNA was detected by 1.2% agarose gel electrophoresis. Phusion® High‐Fidelity PCR Master Mix with GC Buffer (New England Biolab) was used to amplify the V3 and V4 hypervariable regions of 16S rDNA using the following primers (5′–3′): 338F (5′‐ACTCCTACGGGAGGCAGCA‐3′) and 806R (5′‐ GGACTACHVGGGTWTCTAAT‐3′). All samples were sequenced using the paired‐end strategy on Illumina platform. After library establishment, the microbiome bioinformatics was performed with QIIME 22019.4 (Bolyen et al., 2019) with slight modifications according to the official tutorials (https://docs.qiime2.org/2019.4/tutorials/). The raw reads were filtered, merged, demultiplexed for quality control and clustered into operational taxonomic units (OTUS) using a 97% similarity cut‐off from UCLUST. Finally, the ribosomal database project (RDP) was used to analyze the taxonomy of each OTUs.

### 2.11 Statistical analysis

Statistics analyses were performed using GraphPad Prism software (version 7.0). For experiments that presented normal distributions and equality of variances, one‐way ANOVA was employed; otherwise, non‐parametric analyses were utilized for one‐factor analyses. All data represent the mean ± SD, the *p* < .05 were regarded as statistically significant.

## 3 RESULTS

### 3.1 Transcriptome sequencing results of PCOS patients and normal subjects

Following this, the analysis of single‐cell transcriptomic data was performed in our experiment. There were 2417 genes significantly upregulated and 1286 genes significantly downregulated between PCOS patients and control people. Subsequently, KEGG enrichment analysis was performed to explore the underlying mechanisms of PCOS. As depicted in figure 1, AMPK signaling pathway, Endocrine resistance, HIF‐1 signaling pathway, and Synaptic vesicle cycle are enriched by KEGG Pathway analysis on upregulated genes. Metabolic pathway, Peroxisome, Fatty acid metabolism, and Rap1 signaling pathway are enriched by KEGG Pathway analysis on downregulated genes. Our results showed that the pathways might have a role in the occurrence and development of PCOS.

### 3.2 Potential active ingredients of *Moringa oleifera* leaves

A total of 230 chemical constituents of MOL were retrieved through the initial literature search, and 98 potentially active ingredients were obtained after intestinal absorption and drug‐like screening (Table 1).

**TABLE 1:** Potential active ingredients of *Moringa oleifera* leaves.

Number	Name	Number	Nam
MOL1	Isorhamnetin	MOL2	Resveratrol
MOL3	Vanillin	MOL4	Niaziminin B
MOL5	Niazimin A	MOL6	Niazimicin
MOL7	1,7‐Dihydroxy‐2,3‐methylenedioxyxanthone	MOL8	Methyl myristate
MOL9	Tianshic acid	MOL10	Dibutyl sebacate
MOL11	Quinine	MOL12	Glyceryl linolenate
MOL13	Methyl linolenate	MOL14	Methyl nonanedioate
MOL15	Vicenin‐2	MOL16	Apigenin
MOL17	Kaempferol	MOL18	Quercetin
MOL19	Luteolin	MOL20	Ellagic acid
MOL21	Eugenol	MOL22	Coumarin
MOL23	Ethyl citrate	MOL24	6‐Hydroxykaempferol
MOL25	Scutellarein	MOL26	Rhamnetin
MOL27	Myricetin	MOL28	5,7,2′,5′‐Tetrahydroxyflavone
MOL29	Protocatechuic acid	MOL30	Gallic acid
MOL31	Neoxanthin	MOL32	Lutein
MOL33	Ricinoleic acid	MOL34	4‐aminobenzoic acid
MOL35	13‐hydroxy‐9,11,15‐octadecatrienoic acid	MOL36	Methyl hexadecanoate
MOL37	Padmatin	MOL38	Orobol
MOL39	Parinaric acid	MOL40	Palmitoleic acid
MOL41	Naringenin	MOL42	Hesperetin
MOL43	9‐hydroxy‐10,12,15‐octadecatrienoic acid	MOL44	*p*‐Coumaric acid
MOL45	Ferulic acid	MOL46	Caffeic acid
MOL47	Isoferulic acid	MOL48	Sinapinic acid
MOL49	o‐Coumaric acid	MOL50	4‐(1‐Oxopentyl)‐methyl ester, Benzoic acid
MOL51	2‐Monolinolein	MOL52	ρ‐Coumaric acid
MOL53	Adenosine	MOL54	Diethyl phthalate
MOL55	p‐Hydroxybenzoic acid	MOL56	Catechol
MOL57	Pyrogallol	MOL58	Resorcinol
MOL59	Pentadecanoic acid	MOL60	Vanillic acid
MOL61	Galactoarabinan	MOL62	Daidzein
MOL63	Genistein	MOL64	Syringic acid
MOL65	Cirsilineol	MOL66	*n*‐Pentadecanal
MOL67	2,6‐di‐tertbutyl‐4‐methylphenol	MOL68	Phenol
MOL69	Linalool	MOL70	2′‐Hydroxygenistein
MOL71	Pterygospermin	MOL72	Benzyl isothiocyanate
MOL73	Ascorbic acid	MOL74	Palmitic acid
MOL75	Hydroperoxyoctadecatrienoic acid	MOL76	Trihydroxyoctadecadienoic acid
MOL77	3‐Hydroxyoctadecanoic acid	MOL78	Azelaic acid
MOL79	3‐*n*‐Butylphthalide	MOL80	Coronaric acid
MOL81	Sulforaphane	MOL82	3,4,5‐trimethoxycinnamic acid
MOL83	Nicotinamide	MOL84	7‐Hydroxyflavone
MOL85	Zeaxanthin	MOL86	Beta‐Carotene
MOL87	Dimethyl propanedioate	MOL88	Chlorogenic acid
MOL89	Vitamin H	MOL90	Cinnamic acid
MOL91	Oleic acid	MOL92	Citric acid
MOL93	Gentistic acid	MOL94	Moringine
MOL95	Malic acid		

### 3.3 Targets site prediction of the effect of *Moringa oleifera* leaves on polycystic ovary

**FIGURE 2:**
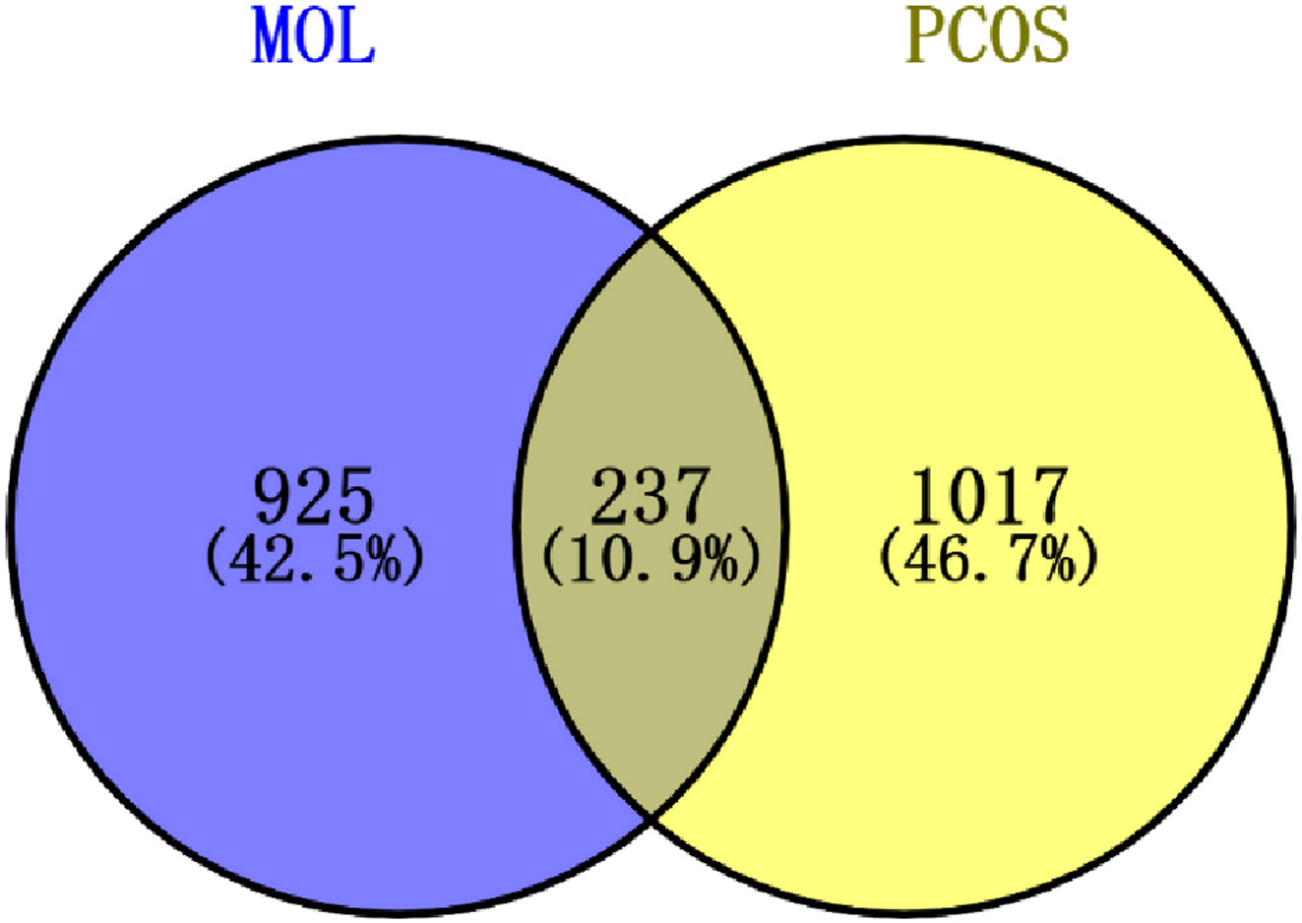
Targets site prediction of the effect of MOL on polycystic ovary.

In total, 5202 targets were screened related to MOL by the SwissTargetPrediction database, and de‐duplicated reads were obtained from 1162 targets. At the same time, we retrieved the PCOS 4982 targets from the Genecards database (https://www.genecards.org/). The target with a relevance score greater than 10 was selected as the prediction target of PCOS. Through Venny 2.1.0 platform, 237 intersection targets of MOL and PCOS were obtained, as shown in Figure 2.

### 3.4 Protein interaction network analysis

**FIGURE 3:**
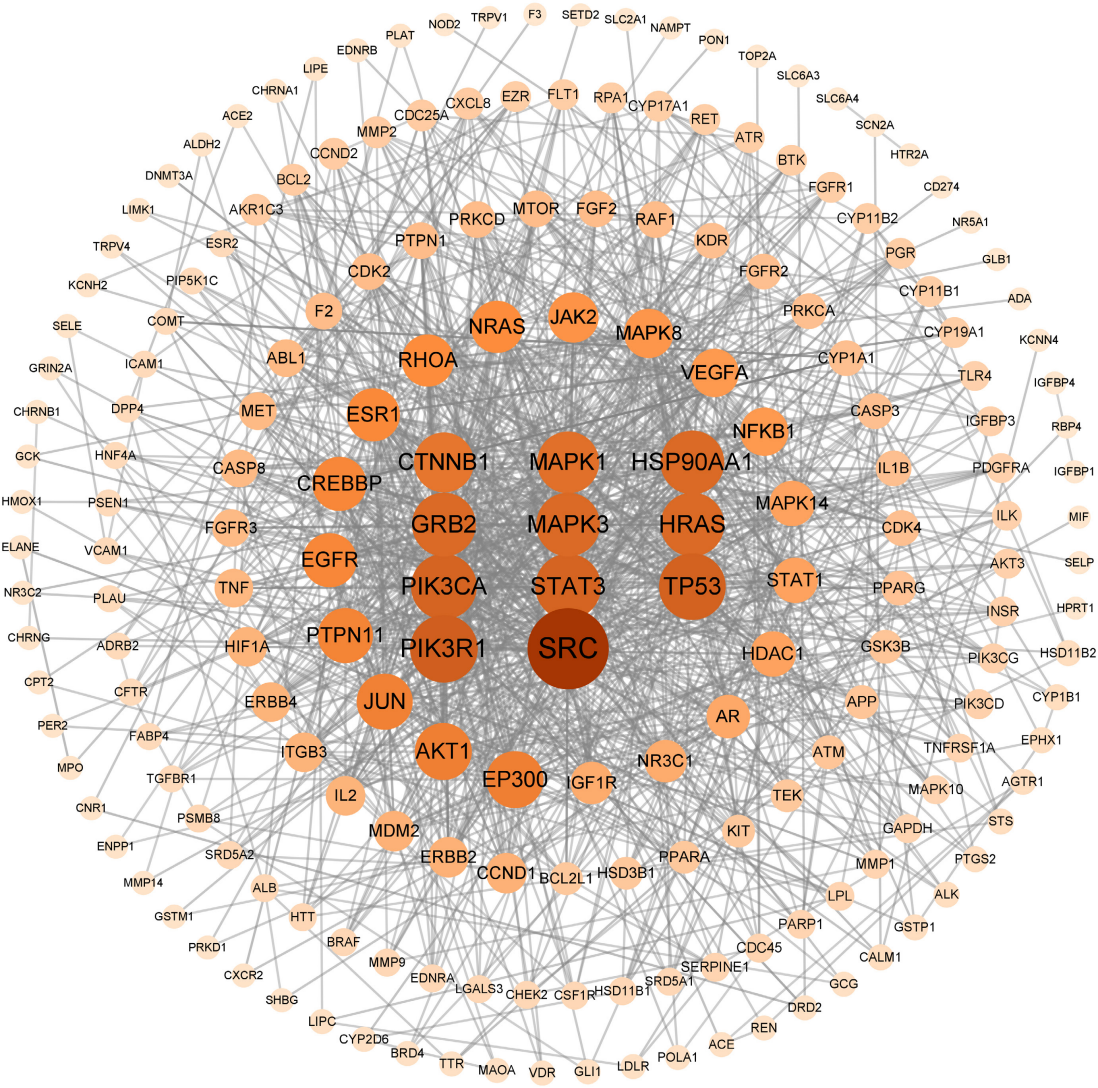
MOL‐PCOS target PPI network.

The common target was imported into STRING database 11.5, and the protein interaction information was obtained (hide disconnected nodes in the network). The PPI network diagram is obtained by using cytocape 3.9.1, as shown in Figure 3. The network consists of 197 nodes and 1070 sides. The results of protein topology analysis showed that SRC, PIK3R1, TP53, PIK3CA, STAT3, HSP90AA1, GRB2, MAPK3, HRAS, MAPK1, and other targets ranked high, indicating that these targets play an important role in the treatment of PCOS.

### 3.5 GO biological process and KEGG pathway enrichment analysis

**FIGURE 4:**
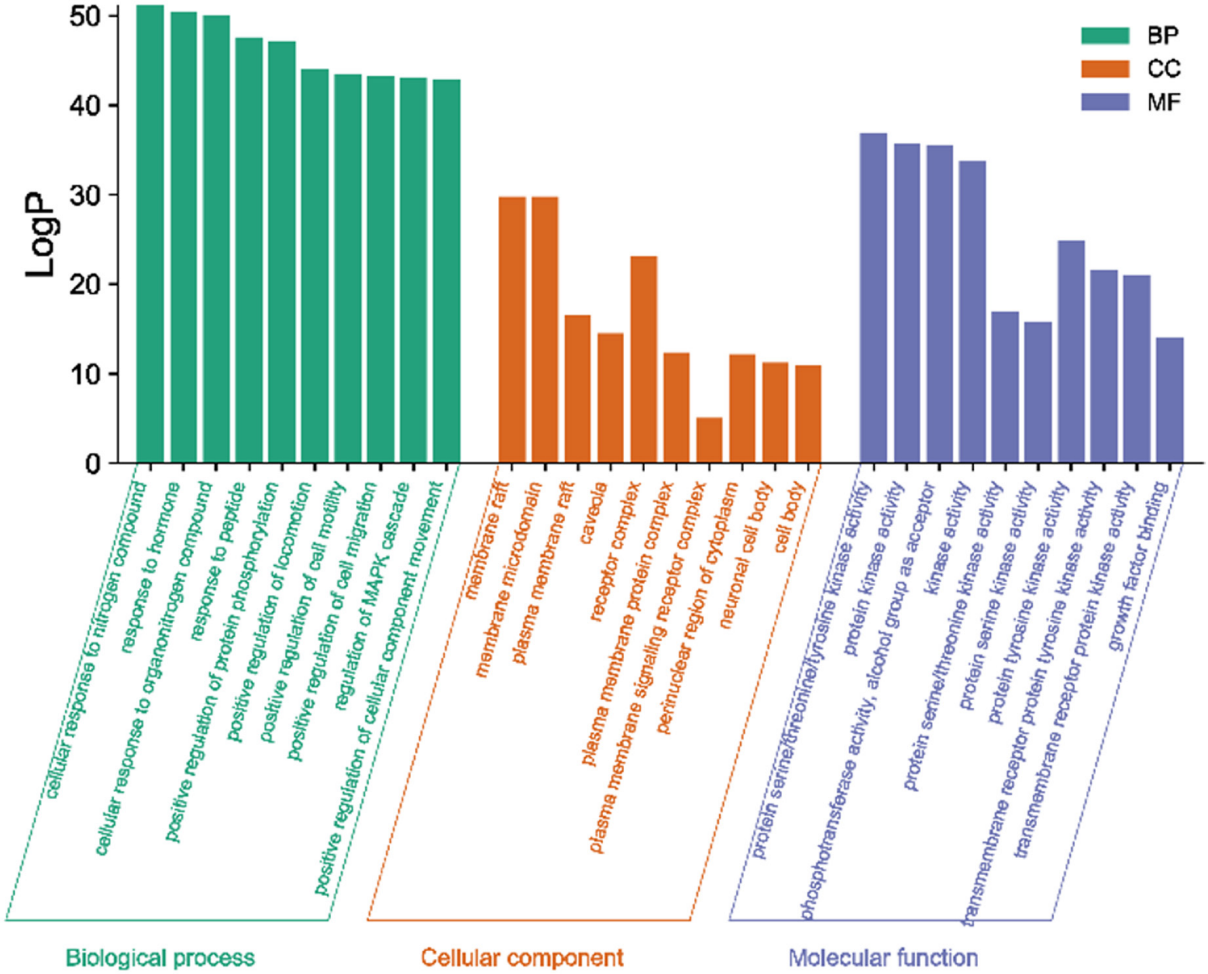
GO biological function enrichment analysis of MOL‐PCOS target.

**FIGURE 5:**
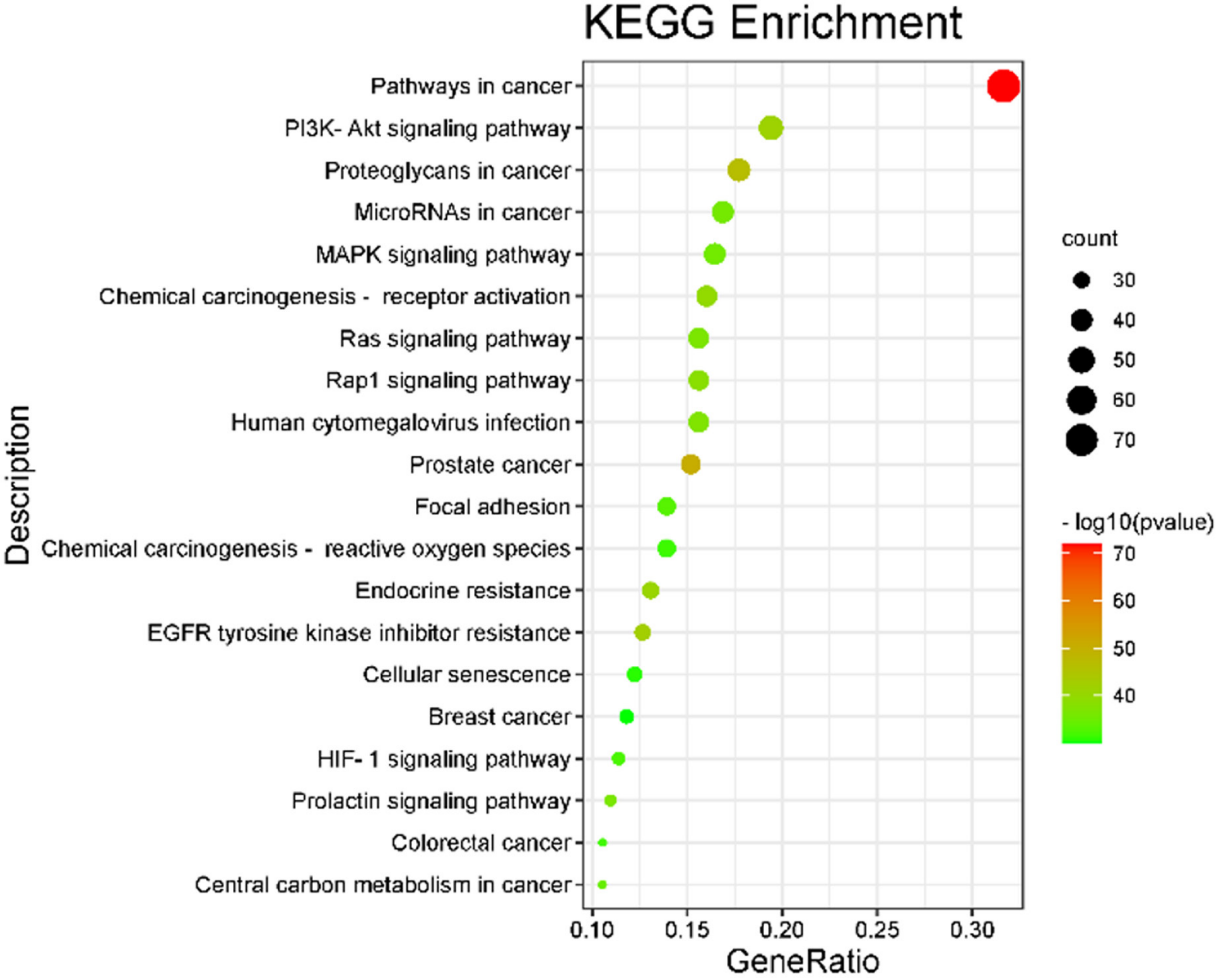
KEGG enrichment analysis bubble chart.

A total of 237 action targets were imported into the Metascape database for bioinformatics analysis. The results include 2326 biological processes (BP), 155 cell composition (CC), and 246 molecular functions (MF). For each category, select the top 10 items for visual analysis, as shown in Figure 4. The results showed that the intervention of *Moringa oleifera* in the biological process of PCOS mainly included cellular response to nitrogen compound, response to hormone, and cellular response to organonitrogen compound. Cell composition mainly includes membrane raft, membrane microdomain, plasma membrane raft. Molecular functions include protein serine/threonine/tyrosine kinase activity, protein kinase activity, phosphotransferase activity, alcohol group as acceptor, etc. A total of 209 signal pathways were obtained by enrichment of KEGG signal pathways, as shown in Figure 5 According to *p* < .01, the first 20 representative pathways were selected as important pathways for visual analysis. The pathways with more enriched targets were pathways in cancer, PI3K‐Akt signaling pathway, proteoglycans in cancer, etc.

### 3.6 Construction of component‐target‐pathway network in *Moringa oleifera* leaf

**FIGURE 6:**
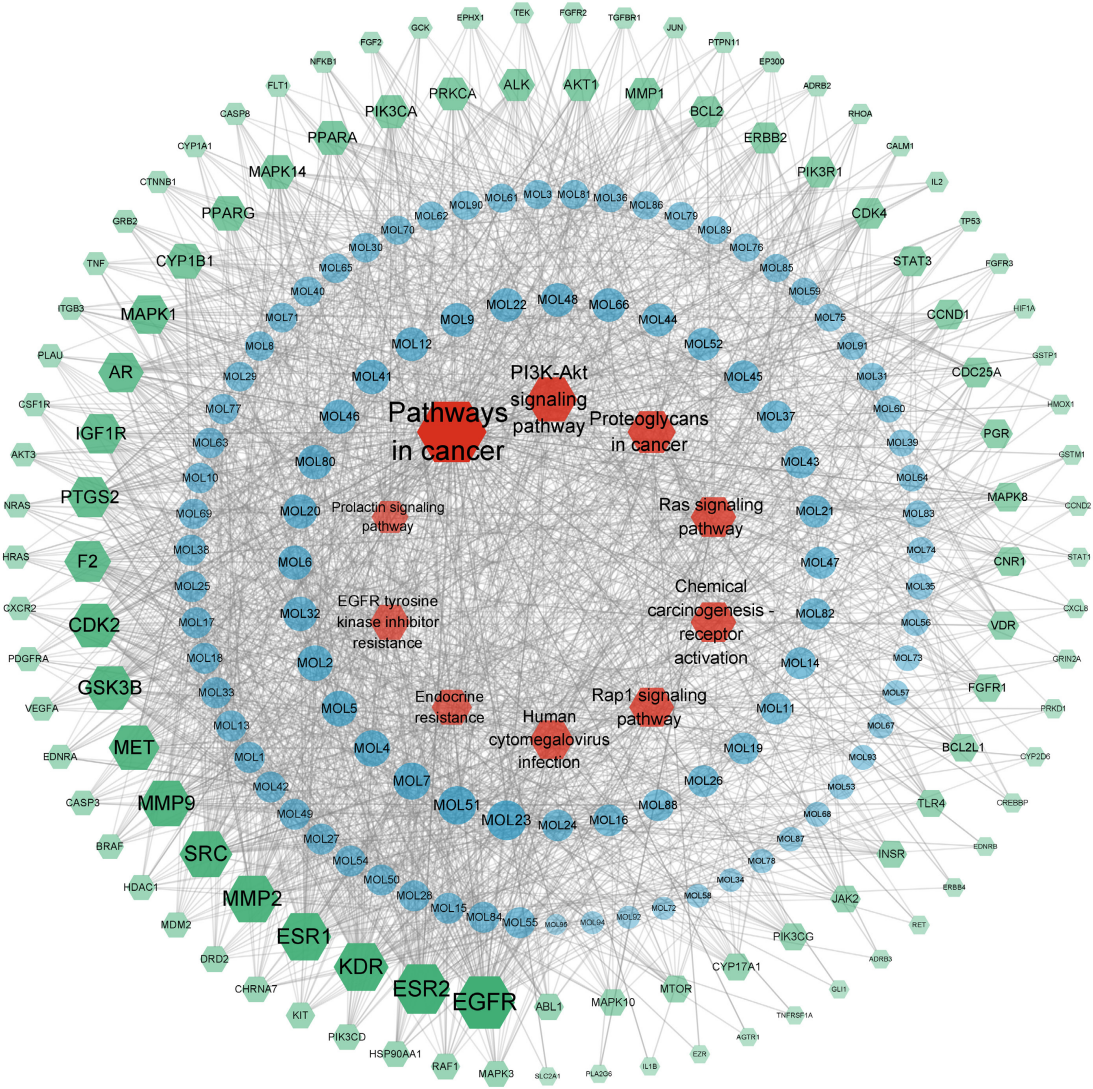
Component‐target‐pathway diagram of MOL in the treatment of PCOS.

Using the built‐in tools of Cytoscape 3.9.1 to analyze the topological parameters of the target network of the intervention of MOL on PCOS, the core components were obtained. As shown in Figure 6, this network is composed of 215 nodes and 1882 edges. The red node represents the top 10 signal pathways of KEGG (removed prostate cancer), the blue node represents the active components of the leaves of Moringa, the green node represents the potential target, and the connecting line represents the interaction among the three. The larger the node area and the darker the color in the figure, the greater the impact on PCOS. Among them, the degree value of MOL23 (ethyl citrate) is 30, the betweenness centrality is 0.017, and the closeness centrality is 0.443. It is predicted that ethyl citrate is the main component of the intervention of MOL on PCOS, followed by MOL51 (2‐Monolinolein) (the degree of connection is 28, the betweenness centrality is 0.016, and the closeness centrality is 0.436) and MOL7 (1,7‐Dihydroxy‐2,3‐methylenedioxyxanthone) (the degree value is 26, the betweenness centrality is 0.015, and the closeness centrality is 0.438). It indicates that the mechanism of the treatment of PCOS by MOL is based on the synergism of multi‐component, multi‐gene, and multi‐target.

### 3.7 Molecular docking screening

**FIGURE 7:**
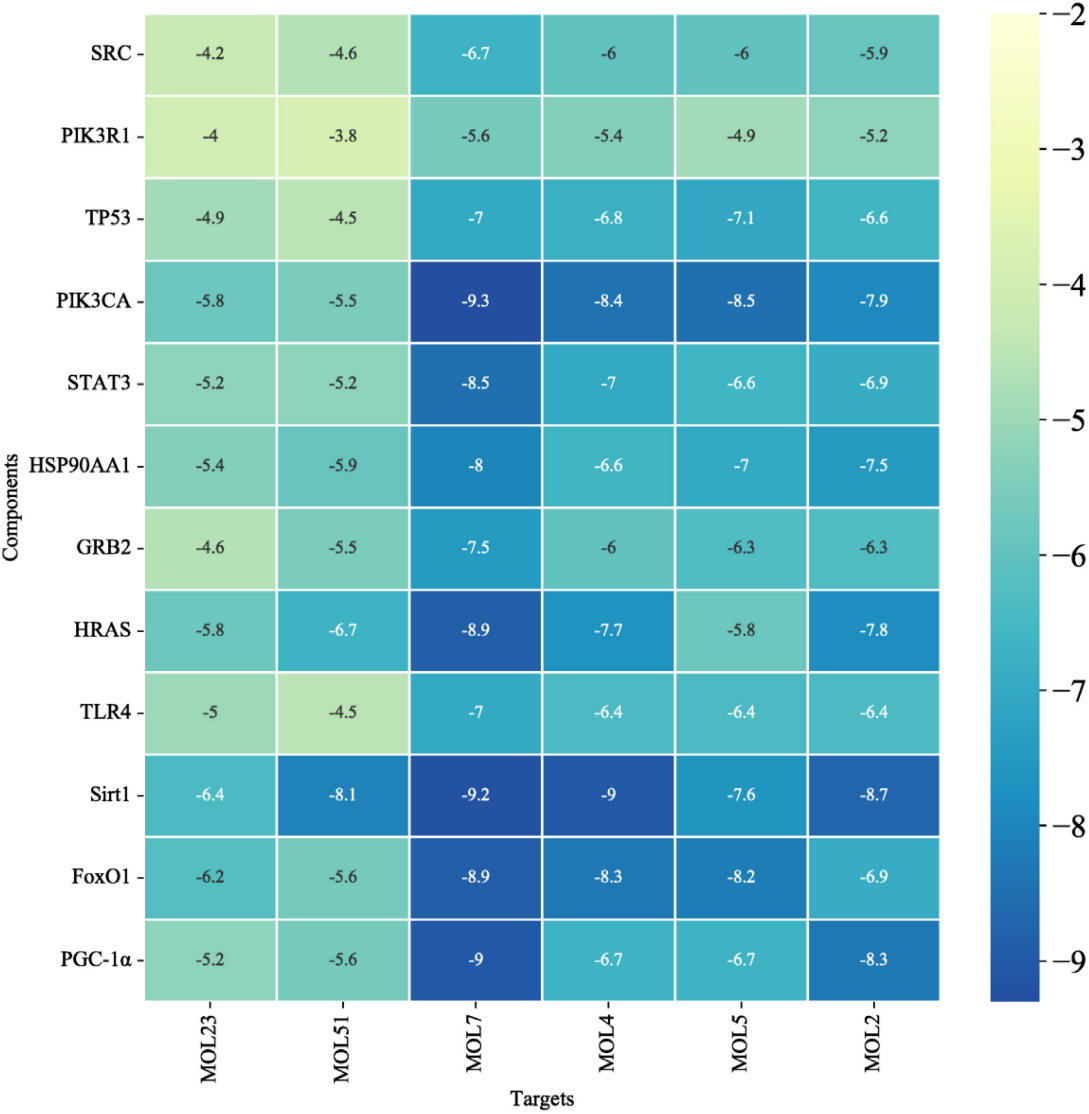
Molecular docking results.

**FIGURE 8:**
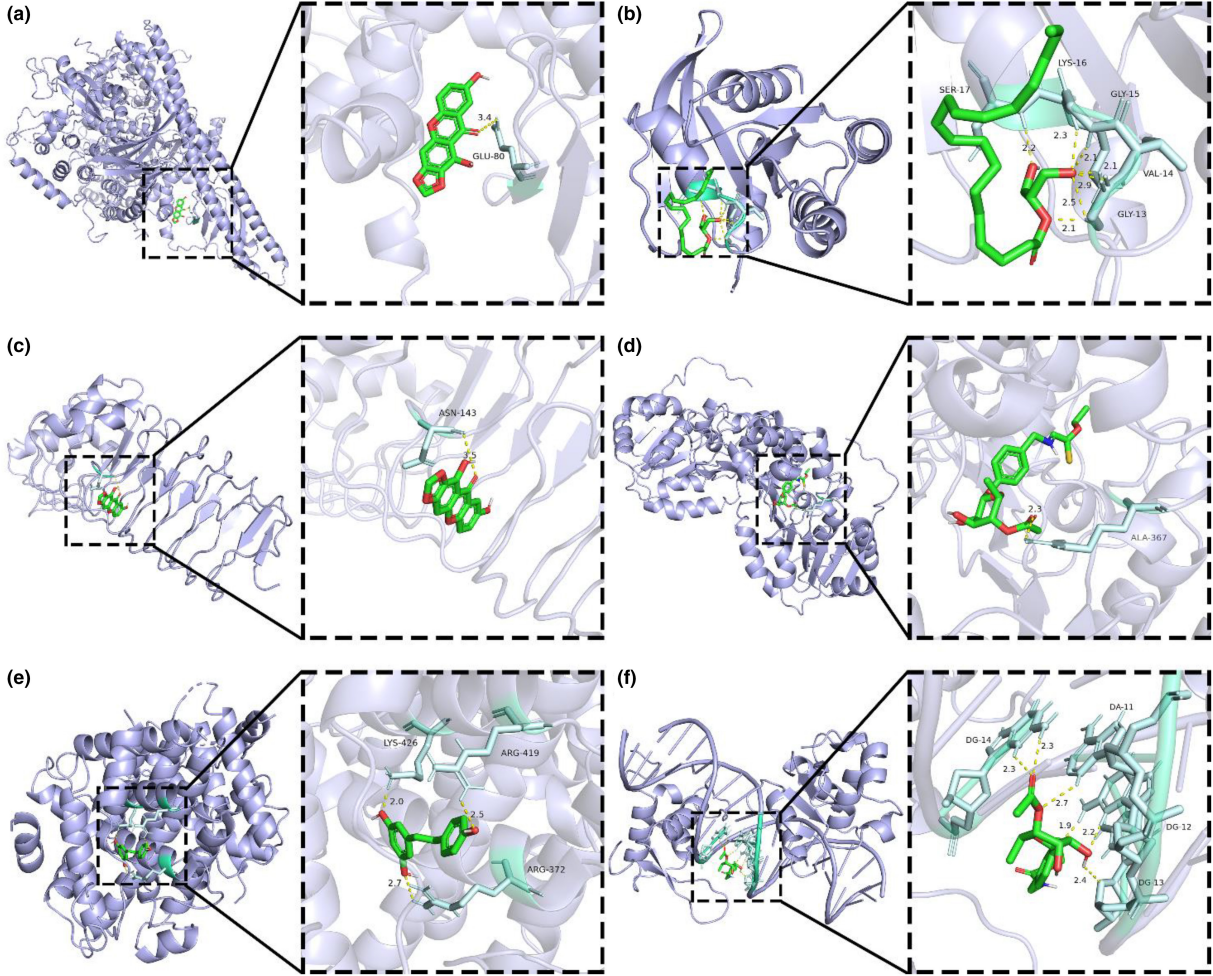
Schematic diagram of docking results of some core compounds: (a) 3D model of PIK3CA (PDBID:4OVU) crystal structure docking. (b) 3D model of HRAS (PDBID:4XVR) crystal structure docking. (c) 3D model of TLR4 (PDBID:6Z62) crystal structure docking. (d) 3D model of Sirt1 (PDBID:4I5I) crystal structure docking. (e) 3D model of FoxO1 (PDBID:4LG0) crystal structure docking. (f) 3D model of PGC‐1α (PDBID:7E2E) crystal structure docking.

Six core compounds were molecularly docked with 11 core targets SRC (PDBID:1O43), PIK3R1 (PDBID:1QAD), TP53 (PDBID:5MF7), PIK3CA (PDBID:4OVU), STAT3 (PDBID:6NJS), HSP90AA1 (PDBID:3RLQ), GRB2 (PDBID:1JYU), HRAS (PDBID:4XVR). TLR4 (PDBID:2Z62), Sirt1 (PDBID:4I5I), FoxO1 (PDBID:4LG0), and PGC‐1α (PDBID:7E2E). Seventy‐two groups of receptor‐ligand docking results were finally obtained. Among the 72 groups of receptor‐ligand results, there were 63 groups with affinity < − 5 kcal · mol^−1^. Among them, the lowest docking score is PIK3CA −1,7‐dihydroxy‐2,3‐methylenedioxythone, with a score of −9.3 kcal · mol^−1^, and the highest docking score is PIK3R1‐2‐mononolein, with a score of −3.8 kcal · mol^−1^, which indicates that the screened potential core compounds may have good binding activity with the core target. See Figure 7 for molecular docking results and Figure 8 for a schematic diagram of docking results of some core compounds.

### 3.8 Supplementation *Moringa oleifera* leaves improve polycystic ovarian morphology, estrous cycle, and sex hormone

**FIGURE 9:**
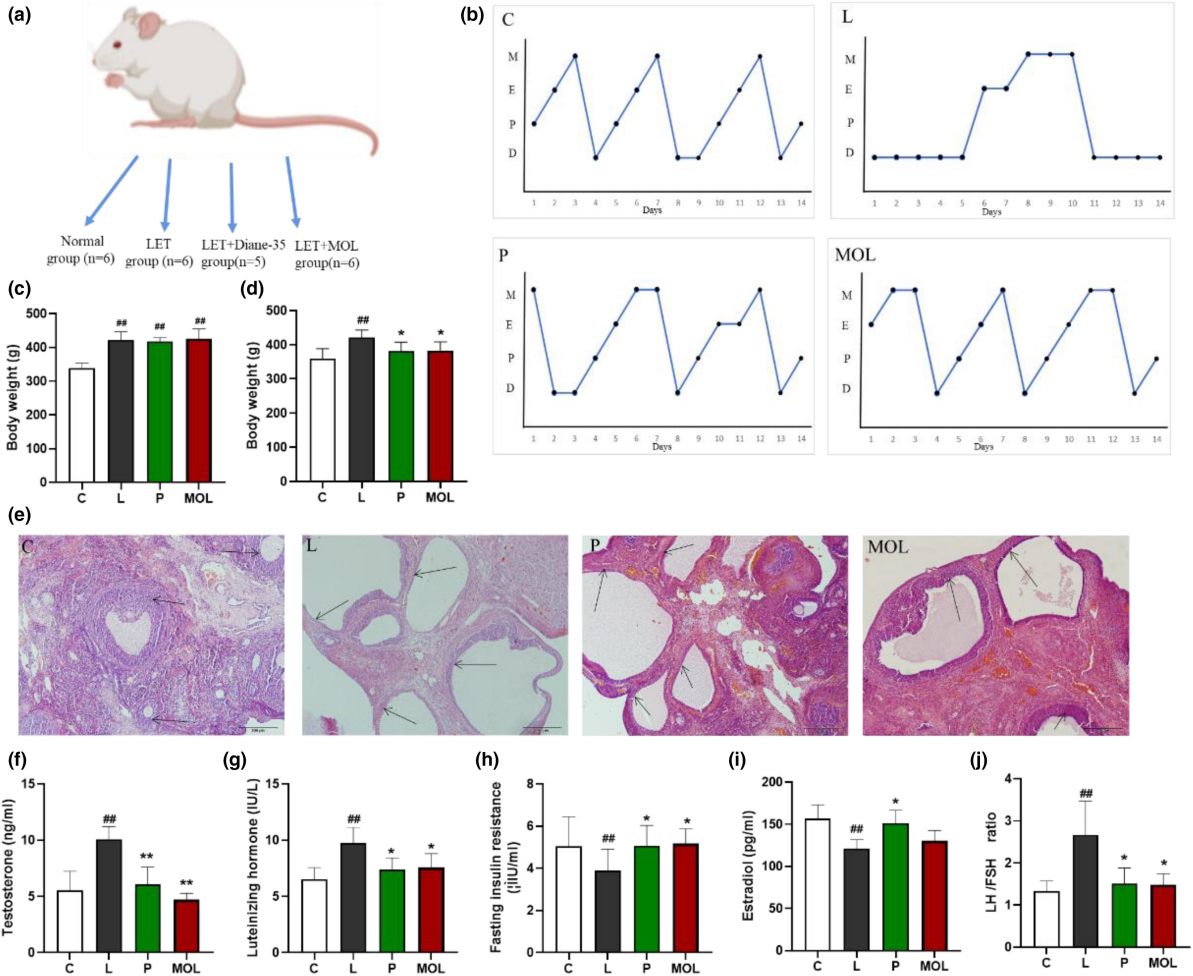
LET‐induced vaginal smear, ovarian morphology, and reproductive hormone level alterations in conventional and MOL‐treated rats. (a) Schematic representation of the above experimental design. (b) Estrous cycle examination, (c) body weight before treatment, (d) body weight after 7 weeks treatment, (e) H&E staining of representative ovaries. Scale bar = 200 μm. (f) Serum T level, (g) serum LH levels, (h) serum FSH levels, (i) serum E2 levels, (j) ratio of LH/FSH. Results of vaginal smear. (D) Diestrus, (P) Proestrus, (E) Estrus, (M) Metestrus. (*p < .05, ** *p* < .01 vs. control; #*p* < .05, ##*p* < .01 vs. L group.)

In order to take a deeper insight into the potential molecular mechanism of PCOS, we conducted further validation in animal models. As shown in Figure 9b,e, the estrous cycle and ovarian morphology of rats in the normal group were normalized when compared with the L group. Simultaneously, the L group showed typical polycystic changes, such as estrous cycle disorder, which basically maintained at interestrus, and increased vesicles, atretic follicles, and thinning of granulosa cell layer. The results are shown in Figure 9b,e, the MOL supplementation improves the ovarian morphology and estrous cycle of rats, with decreasing atretic follicle, vesicle number and size decrease, and enlarged granulosa cell, a thickened granulosa cell layer, and the disorder estrous cycles restored. There were no significant differences in the weight among L, P, and MOL groups, but both had increased weight when compared to the control group (*p* < .01) (Figure 9c). However, the body weight of both treated groups (P, MOL) significantly decreased, as compared to model rats (*p* < .05) (Figure 9d). Furthermore, the serum testosterone and luteinizing hormone concentration were higher in the L group (10.08 ± 0.4637 mmoL/L, 9.763 ± 0.5672 mmoL/L) compared with the C group (5.541 ± 0.6969 mmoL/L, 6.54 ± 0.4188 mmoL/L; *p* < .01). And the estradiol and follicle‐stimulating hormone was lower in the L group (121 ± 4.415 mmoL/L, 3.882 ± 0.4185 mmoL/L) than those in the C group rats (156.7 ± 6.673 mmoL/L, 5.047 ± 0.5703 mmoL/L; *p* < .01). The LH/FSH was significantly increased in the L group rats as compared with the C group rats (*p* < .01). Following the MOL intervention, compared with the L group rats, the concentration of testosterone and luteinizing hormone was obviously decreased, the estradiol and follicle‐stimulating hormone was increased, but without significant difference, the LH/FSH was significantly decreased (Figure 9f–j).

### 3.9 Supplementation *Moringa oleifera* leaves could ameliorate insulin resistance in LET‐induced rats

**FIGURE 10:**
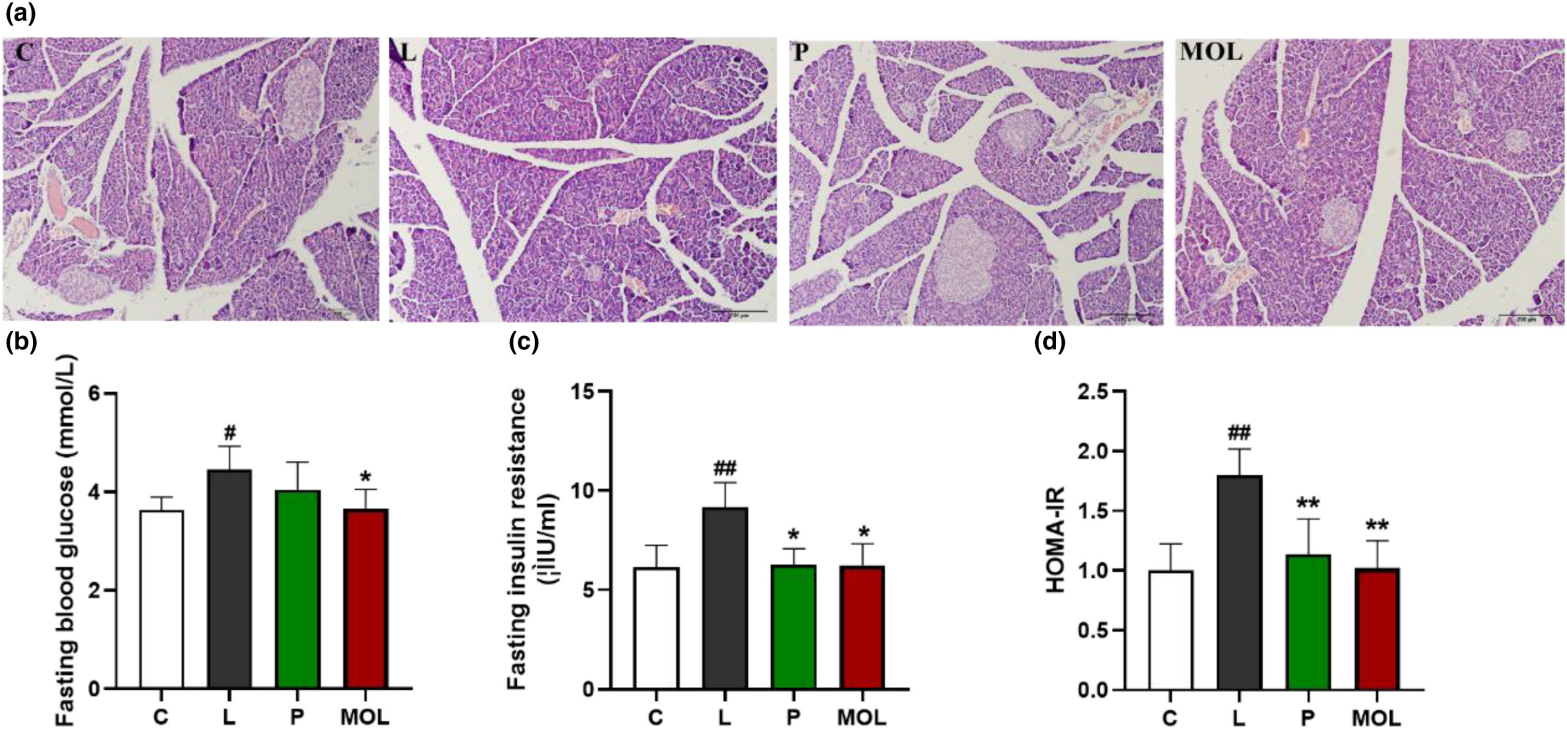
Results of pancreas tissues morphology and the glucose metabolism changes, (a) H&E staining of representative pancreas, (b) serum FBG levels, (c) serum FINS levels, (d) the level of HOMA‐IR. (*p < .05, ** *p* < .01 vs. control; #*p* < .05, ##*p* < .01 vs. L group.)

In order to explore whether MOL could ameliorate insulin resistance in PCOS‐like model rats. As showcased in Figure 10a, the H&E stained sections of the pancreas showed that islet morphology appeared normal in the C group. This difference is reflected in the looser and more variable organization of cells within islets, and the numbers of islets were reduced, cell with enlarged, irregular nuclei, cells exhibiting abnormal in the L group. Interestingly, supplementation of MOL, H&E of islets indicated that the MOL‐treated animals had an obviously greater number and morphology of total islets than the PCOS‐like rat. And the MOL group rats also showed uniform size, regular shape, and clear boundaries of the pancreas. Then, we observed the changes of FBG and FINS in serum and calculated the insulin resistance index. As shown in Figure 10b–d, compared with the C group, the serum FBG, FINS, and the HOMA‐IR were obviously increased in the L group (*p* < .01, *p* < .05). Following the MOL treatment, the FINS, FBG, and HOMA‐IR were markedly decreased when compared with the L group.

### 3.10 Supplementation *Moringa oleifera leaves* decreased the oxidative stress in the ovary of LET‐induced rats

**FIGURE 11:**
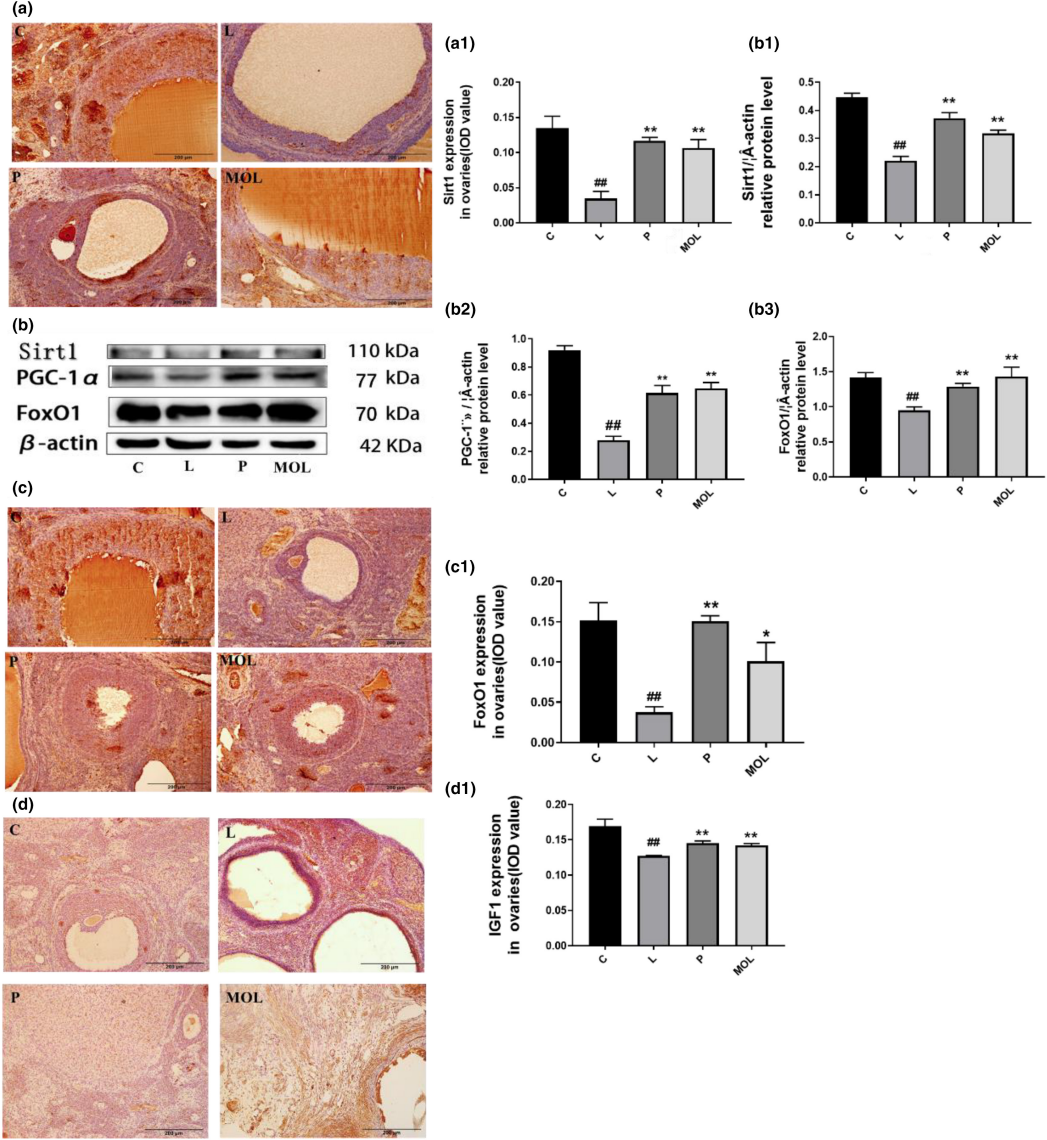
Effect of MOL treatment on the oxidative stress expression in the ovary of PCOS‐like rats. (a, c, d) IHC analysis of Sirt1/FOX01/IGF1 protein expression on the ovaries of PCOS‐like rats after different treatments. (b) Western bolt analysis of the effects of MOL treatment on the protein levels of Sirt1, PGC‐1α, and FOX01 expression in the ovarian from PCOS‐like rats (*p < .05, **p < .01 vs. control; #*p* < .05, ##*p* < .01 vs L group.)

The initiation and progression of PCOS are linked to oxidative stress, an imbalance of free radicals and antioxidants. The synthesis of large amounts of oxygen free radicals can cause lipid peroxidation damage to ovarian tissues and insulin pancreatic islet β cells, which can cause PCOS and its insulin resistance. Therefore, the expression of Stir1, FoxO1, and PGC‐1α was detected by immunohistochemistry and western bolt to evaluate the effects of MOL on anti‐oxidation. Figure 11a,c show that Stir1, FoxO1 is mainly distributed in the nucleus of granulosa cells in ovarian tissue. And the expression of Stir1, PGC‐1α, FoxO1, and IGF1 was significantly downregulated in LET‐induced PCOS rats when compared with the normal group rats (Figure 11a1,b1–b3,c1,d1) (*p* < .01). However, following the MOL supplementation, the expression of Stir1, PGC‐1α, FoxO1, and IGF1 was markedly upregulated than LET‐induced PCOS rats (Figure 11a1,b1–b3,c1,d1).

### 3.11 Supplementation *Moringa oleifera* leaves improve gut barrier function and gut permeability

**FIGURE 12:**
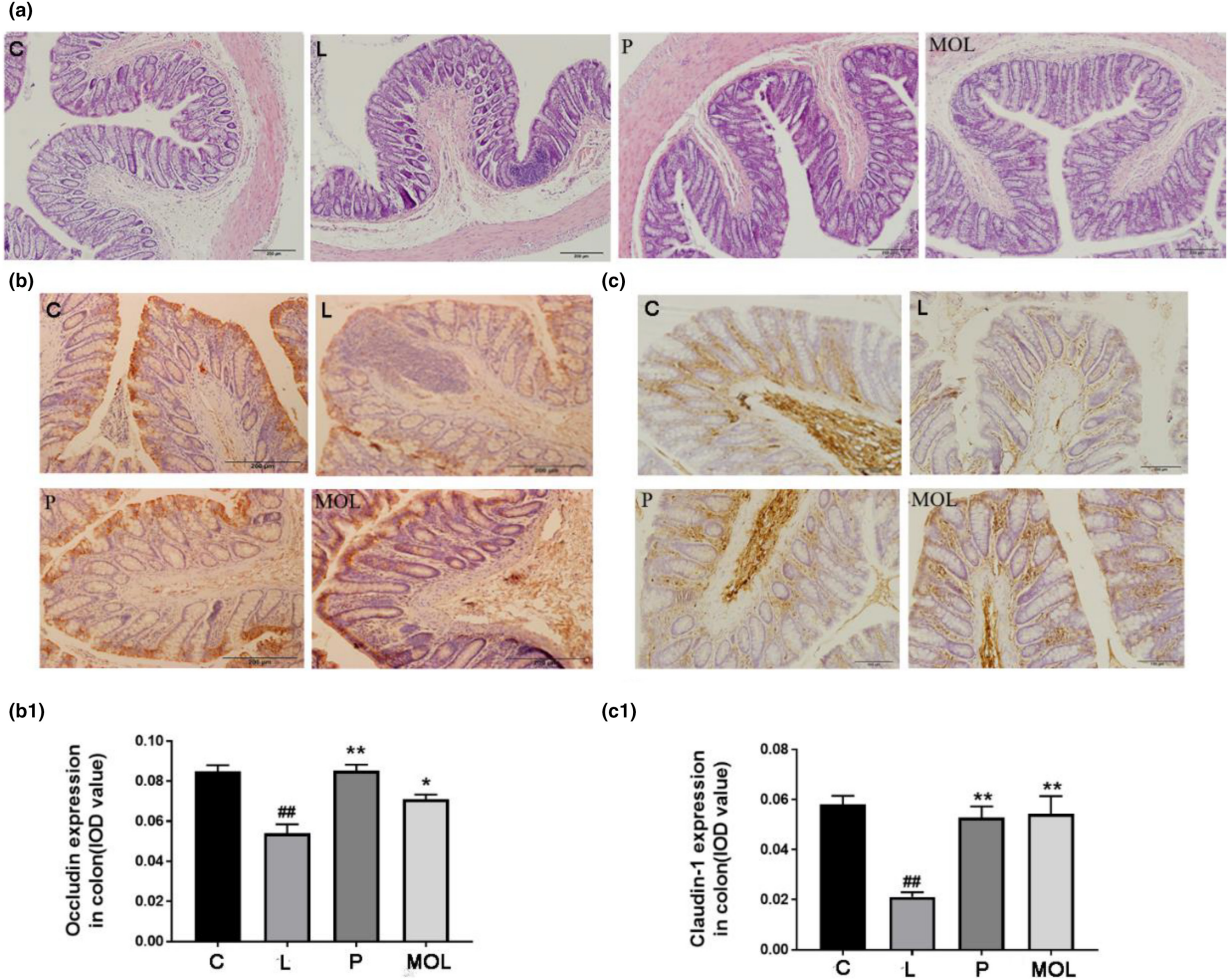
Effect of MOL treatment on the gut barrier function and permeability expression in the colon of PCOS‐like rats. (a) H&E staining of representative colon, (b, c) Effect of MOL treatment on the occludin, claudin‐1 expression levels in the colon tissues from PCOS‐like rats. (*p < .05, ** *p* < .01 vs. control; #*p* < .05, ##*p* < .01 vs., L group.)

To evaluate the effects of MOL on the intestinal function of PCOS‐like rats. Representative HE staining images of colon sections are shown (Figure 12a), in the L group was mostly focused to the mucosa with loss of goblet cells, crypt damage, mucosal ulceration, and accompanying an inflammatory infiltrate in the submucosa, compared with the normal group. Following the MOL supplementation, gross mucosal architecture is intact, including the mucosal, submucosal, and smooth muscle architecture appeared normal, without infiltration of the submucosa. Furthermore, immunohistochemistry revealed that rats with letrozle induction showed decreased expression of claudin‐1 and occludin compared with the normal group (Figure 12b,b1,c,c1) (*p* < .01). After MOL treatment, the expression of occludin and claudin‐1 markedly upregulated when compared with the L group rats, which showed the barrier function and intestinal permeability markedly improved (Figure 12b,b1,c,c1).

### 3.12 Supplementation *Moringa oleifera* leaves changed the relative abundance of multiple intestinal flora

**FIGURE 13:**
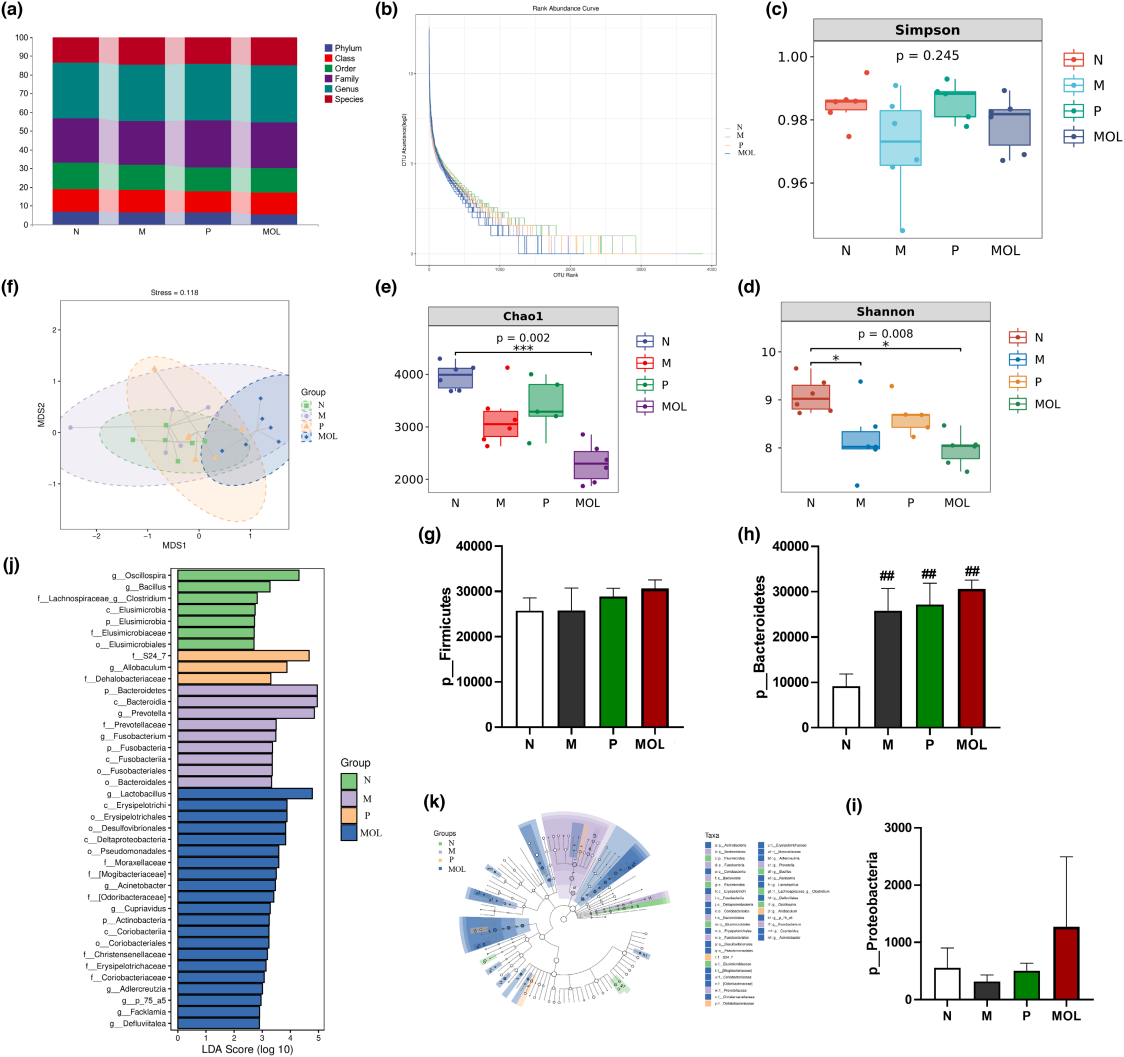
Taxonomic profile of the gut microbiomes of the samples collected from the four groups. (a) The OUT numbers of Phylum, Class, Order, Family, Genus, (b–e) the microbial diversity and richness of the microbiota in the feces. (f) Beta diversity of the microbiota in the feces. (g–i) Relative abundance of gut microbial species at the phylum levels in the feces of rats. (j, k) Linear discriminant analysis (LDA) effect size (LEfSe) results on gut microbiota (*p < .05, ** *p* < .01 vs. control; #*p* < .05, ##*p* < .01 vs. L group).

It is well known that PCOS is associated with intestinal flora, to measure bacterial composition in the gut after supplementation MOL of PCOS‐likes rats. The abundance and composition of microbiota were analyzed by high‐throughput sequencing of 16S rRNA genes (V3‐V4 region) with Illumina MiSeq (San Diego, CA, USA) in the fecal samples. Initially, by analyzing the number of OTUs at each classification level of phylum, class, order, family, genus, and species. We found that species differences are mainly concentrated at the genus level (Figure 13a). *α*‐Diversity was measured by the Chao index, Shannon index, and Simpson index in the intestinal microbiota communities. The result showed that the microbial diversity and richness were significantly different among the L group and C, P, MOL groups (Figure 13b–e). Simultaneously, the Beta diversity analysis showed a marked difference by the anosim and permanova in four groups (Figure 13f). Next, OTU differences across groups were analyzed using *LEfSe*, many microbial taxa markedly differed among the C group and L, P, MOL groups (Figure 13j,k). Our study found that the bacteria of *Firmicutes Bacteroidetes* and *Protecobacteria* phylum were obviously changed. In the C group, *Firmicutes* accounted for 71.95%, *Bacteroidetes* for 25.584%, and *Protecobacteria* for 1.555%. Compared with the L group, there was an increase in the number of *Firmicutes* and *Protecobacteria*, and decrease in the number of *Bacteroidetes* in the MOL group (Figure 13g–i). We also found that the bacteria of the *Fusobacteria* family and *Fusobacterium* genus and *Fusobacteriia* class and the bacteria of the *Prevotellaceae* family and *Prevotella* genus were increased in the L group when compared with the C group. It was an interesting that the bacteria of the *Lactobacillus* genus and the *Desulfovibrionales* order and *Defluviitalea p_75_a5 Facklamia Clostridium* and *Adlercreutzia* genus, and the bacteria *Christensenellaceae* and [*Odoribacteraceae*] family were over‐represented, while the relative abundance of Fusobacterium, Prevotella was decreased in the MOL group (Figure 13e,f).

### 3.13 Supplementation *Moringa oleifera* leaves ameliorated inflammation in LET‐induced rats

**FIGURE 14:**
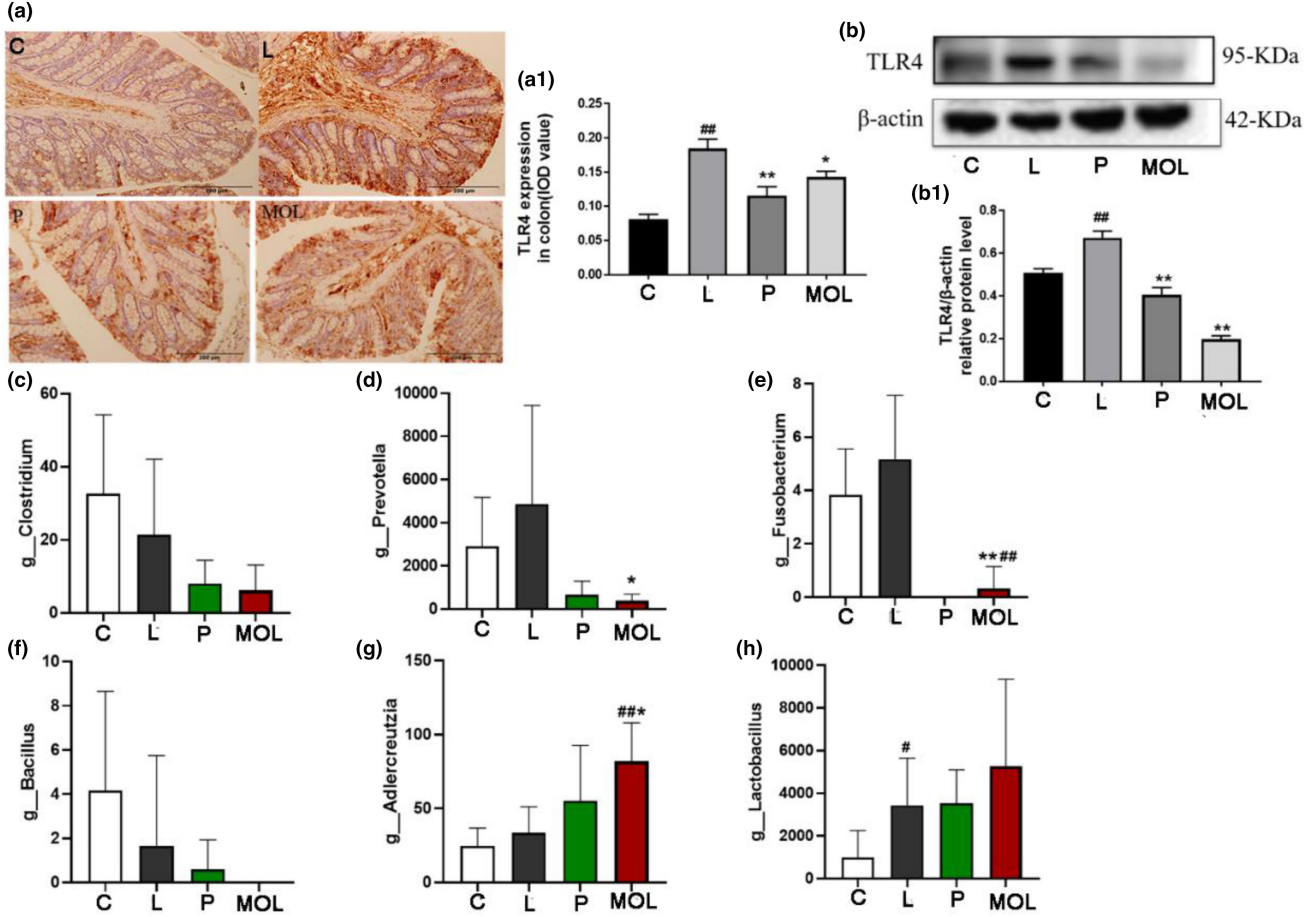
Effect of MOL treatment on the inflammation expression in the ovary, colon, and intestinal flora of PCOS‐like rats. (a) IHC analysis of TLR4 protein expression on the colon of PCOS‐like rats after different treatment. (b) Effect of MOL treatment on the TLR4 expression levels in the ovaries tissues from PCOS‐like rats. (c–h) Effect of MOL treatment on relative abundance of intestinal flora species at the genus levels related to inflammation in the feces of rats. (*p < .05, **p < .01 vs. control; #*p* < .05, ##*p* < .01 vs. L group.)

Patients with PCOS have chronic microinflammation, which is involved in the entire pathophysiological process of the disease. Therefore, in order to determine whether MOL could be anti‐inflammatory in PCOS‐like model rats, we detect the expression of TLR4 on ovary and colon tissues, founding that the expression of TLR4 was higher in LET‐induced PCOS‐like rats than in normal group rats (Figure 14a,a1,b,b1). However, the expression of TLR4 was significantly decreased in the MOL group when compared with the LET‐induced rats (Figure 14a,a1,b,b1). This indicates that MOL has a beneficial effect on PCOS‐like rats, which may be related to the reduction of TLR4.

### 3.14 The gut flora was related with biochemical indices

**FIGURE 15:**
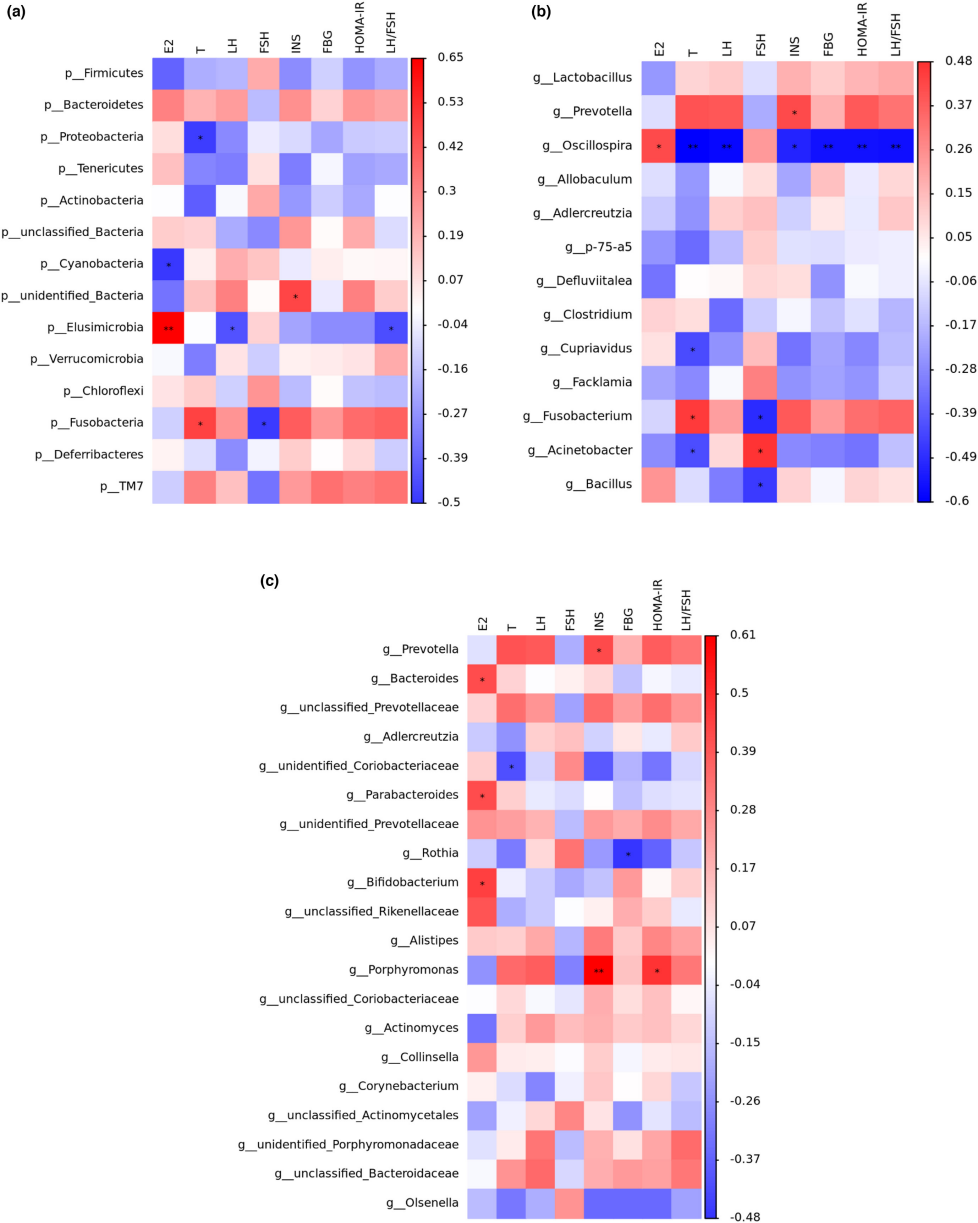
Correlation analyses between relative abundance (%) of microbiota and other related indicators. (a) Relative abundance of the phylum levels and biochemical indices. (b) Relative abundance of the Top 20 genus levels and biochemical indices. (c) Relative abundance of the differential genus levels and biochemical indices. Relative abundance of gut microbial species at the genus levels in the feces of rats.

We performed a correlation analysis for the assessment of relations among hormone profiles and gut microbiota in PCOS and supplementation MOL rats (Figure 15a–c). The abundance of “bad bacteria” including Fusobacteria, Fusobacterium, Porphyromonas, and *Prevotella* was positively correlated with the levels of luteinizing hormone, testosterone, LH/FSH, insulin, and HOMAR‐IR respectively. Meanwhile, they were negatively associated with E2, FSH. Interestingly, after supplementation of MOL, the abundances of beneficial bacteria (*Bifidobacterium*, *Acinetobacter*, *Oscillospira*, *Cupriavidus*, *Clostridium*, *Parabacteroides*, *Bacteroides*, and *unidentified_Coriobacteriaceae*) exhibited positively correlated with FSH, E2, respectively. However, they were negatively associated with the levels of T, LH, LH/FSH, insulin, and HOMAR‐IR. Taken together, there were close interactions and correlations among sex hormones, glucose metabolism, and gut microbiota were closely correlated.

## 4 DISCUSSION

The growth and development of organisms, the renewal of histiocyte, the maintenance of basic life activities (such as heartbeat, respiration, body temperature, and keeping the brain awake), and labor all need to obtain essential nutrients from the external environment. These essential nutrients include sugars, lipids, proteins, water, inorganic salts, and vitamins. Sugar, fat, and protein can gradually release the energy needed to maintain basic life activities and labor during the process of oxidative decomposition in organisms, so sugar, fat, and protein are also known as the three major energy substances. MOL are rich in sugars (14.36 g/100 g), fats (8.08 g/100 g), and proteins (24.2 g/100 g), so they can provide essential nutrients for life activities and complete energy cycle metabolism (Table S1).

*Moringa oleifera* leaf is the most nutritious and utilized part of the *Moringa oleifera* and exerts multiple biological functions effects on antidiabetic, antibacterial, antioxidants, and anti‐inflammatory (Afzal et al., 2021). Oxidative stress, inflammation, obesity, and insulin resistance are frequently tied to PCOS and also described as the risk factors for the development of metabolic syndrome. It is worth mentioning that oxidative stress markers are often associated with increased inflammation (Jamilian et al., 2019). The components in MOL as bifunctional molecule exert antioxidant activity either directly by scavenging ROS or indirectly by inducing the antioxidant response and, therefore, ameliorating the redox status of the cells (Duranti, Maldini, Crognale, Sabatini, et al., 2021).

In the present study, we carried out KEGG enrichment analysis of the differential genes in the whole‐transcriptome sequencing of MII oocytes from PCOS and control women. Simultaneously, our study also made a network pharmacological analysis of the components of MOL and predicted its effect on PCOS. In the results, the enrichment analysis of the intersection target shows that there are multiple pathways involved in the improvement of PCOS by MOL, such as HIF‐1 signal pathway, Endocrine resistance, Steroid hormone biosynthesis, AMPK signal pathway, oxidative stress and inflammation, and other related signal pathways. At the same time, we carried out KEGG enrichment analysis on the differential genes of transcriptome sequencing in the follicles of patients, and found that these differential genes were also involved in multiple pathways. On this basis, we performed molecular docking between the selected core components and the core targets. The molecular docking technology shows that the two have good docking activity. Interestingly, we found that the docking results of TLR4, Sirt1, FoxO1, and PGC‐1α with the core components of MOL were all less than −4.5 kcal · mol^−1^, especially the docking results of Sirt1 with 1, 7‐dihydroxy‐2,3‐methylenedioxyxanthone (MOL7) were −9.2 kcal · mol^−1^. These results indicate that TLR4, Sirt1, FoxO1, and PGC‐1α have strong affinity with core components. Therefore, TLR4, Sirt1, FoxO1, and PGC‐1α may be potential targets for the treatment of PCOS. In order to confirm this idea, we did a further in vivo experimental study. As reported, reducing oxidative stress in PCOS patients, or strengthening their antioxidant defense systems, can be beneficial to decrease and improve the complication of the disease (Zhang et al., 2019). Previous in vivo and in vitro studies showed that MOL supplementation ameliorated mitochondrial oxidative damage and reduces inflammation, and lipid accumulation (Alqahtani & Albasher, 2021; Duranti, Maldini, Crognale, Horner, et al., 2021). Therefore, to further elucidate the interactions between various changes during the development of PCOS, we constructed an animal model of PCOS.

We investigated the effect of supplementation MOL on oxidative stress and inflammation with LTE‐induced PCOS‐like rats. Our experimental results show the level of Sirt1, PGC‐1α, FoxO1, and IGF1 in PCOS‐like rats decreased while MOL could upregulate the expression of Sirt1, PGC‐1α, and FoxO1 in the ovary. As a class III nicotinamide adenine dinucleotide‐(NAD‐)dependent histone deacetylase, silent information regulator 1 (Sirt1) has gained a lot of attention for its role in oxidative stress resistance (Zhang et al., 2017). Peroxisome proliferator‐activated receptor γ coactivator‐1α (PGC‐1α) is an activator in the cell nucleus that regulates various biological actions, it acts as an essential node connecting metabolic regulation, redox control, and inflammatory pathways, and it is an interesting therapeutic target (Rius‐Pérez et al., 2020). In addition, PPAR and HIF‐1 signal pathway was found in our network pharmacological analysis, which is closely related to PGC‐1α and Sirt1. PGC‐1α could activate PPARs to induce mitochondrial gene expression and promote oxidative metabolism (Ventura‐Clapier et al., 2008). Hypoxia‐inducible factor 1‐alpha (HIF‐1α), as a master regulator in the HIF‐1 signal pathway, is a hypoxia‐activated transcription factor that confers protective effects in hypoxic conditions. Studies have reported that Sirt1 deacetylates and stabilizes HIF‐1α through direct interactions (Joo et al., 2015). The forkhead‐box transcription factor O1 (FoxO1) is an important substrate of Sirt1 and has been shown to play a vital role during glycolipid metabolism, insulin resistance, and oxidative stress (Xu & Wang, 2021). Recent studies found that FoxO1 is involved in follicular development through the regulation of apoptosis and proliferation of granulosa cells (Shen et al., 2012). Studies have shown that Sirt1 can activate FoxO1 by decetonylation and anti‐oxidation stress, and the activation of FoxO1 can also increase the expression of Sirt1 (Ronnebaum & Patterson, 2010; Yao et al., 2018). Clearly, Sirt1 activates PGC‐1α and FoxO1 by decetonyaltion, removing ROS caused by oxidative stress, reducing oxidative stress damage, and alleviating inflammation. Related studies have reported that patients with PCOS were accompanied by features of oxidative stress and mitochondrial impairment, which can aggravate insulin resistance (Alam et al., 2021), it is consistent with the insulin resistance of PCOS‐like rats in this study. It is worth mentioning that, following MOL supplementation, the body weight, FBG, FINS, and HOMA‐IR were ameliorated. Since, Sirt1, FoxO1, and PGC‐1α had been shown to inhibit oxidative stress, we evaluated its potential to combat oxidative stress‐related PCOS in MOL. According to reports, IGF1 can enhance the proliferation of ovarian cells, promote progesterone production, and inhibit cell apoptosis (Sirotkin et al., 2019). Research has found that mIGF‐1 protects myocardial cells from oxidative stress through Sirt1 activity. PCOS patients generally have insulin resistance, and IGF1 can enhance pancreatic β cell viability and inhibit apoptosis by activating the Sirt1/PI3K/Akt/FoxO1 pathway, inducing insulin secretion, and alleviating oxidative damage (Vinciguerra et al., 2009). Our experimental results also confirmed that MOL can upregulate the expression of IGF1, thereby improving the symptoms of PCOS. Therefore, we speculated that MOL ameliorated PCOS by alleviating oxidative stress through the Sirt1/FoxO1/ PGC‐1α signaling pathway.

Considering that stress induced the production of pro‐inflammatory cytokine, the inflammatory response is currently considered to be a key link of sex hormone imbalance and insulin resistance in PCOS patients. Toll‐like receptors (TLRs) are innate immune pattern recognition receptors, several studies have shown that TLR4 is involved in inflammatory responses in PCOS (Huang et al., 2021). To further explore whether MOL has anti‐inflammatory on PCOS‐like rats, the TLR4 was detected in ovary and colon tissues, which expression is high in PCOS‐like rats. Interestingly, we found that supplementation MOL downregulated the expression of TLR4, this suggests the effect of MOL on PCOS‐like rats anti‐ inflammatory may be at least partially mediated by the TLR4 pathway. Moreover, Sirt1 is a multifunctional molecule involved in a variety of inflammatory pathways (Wang, Weng, et al., 2020). The study reported here shows that Sirt1 can inhibit the expression of TLR4 induced by TNF‐α in the cell culture system (Yuan et al., 2018). AS reported that patients with low *PGC*‐*1α* present an inflammatory profile whereas patients with high *PGC*‐*1α* exhibit better antioxidant status (Xu et al., 2014). Notably, our experimental results show that the expression of TLR4 was downregulated when the Sirt1 and *PGC*‐*1α* upregulated after MOL intervention, which further illustrates that the Sirt1 and *PGC*‐*1α* are activated to interfere with downstream inflammatory factors and apoptosis, and turn affected PCOS. In addition, TLR4 is one target in the HIF‐1 signal pathway, where neutralizing HIF‐1α weakened the increase in TLR4 in BV‐2 cells in hypoxia condition with downregulation of TNF‐α expression (Riddell et al., 2012). Thus, these data suggest that the inhibitory effect of MOL on TLR4 may be regulated by multiple proteins and complex signal networks.

Additionally, this study also underlines the effect of MOL treatment on the intestinal barrier and intestinal microbiota in the LET‐induced PCOS rats. The integrity of the intestinal barrier is essential for maintaining intestinal permeability and preventing diseases associated with PCOS and its complications. Thus further investigation assessed the intervention efficacy of MOL on the intestinal barrier by mucosa, submucosa, muscular layer and serosa, the thickness of muscle layer, and the number of goblet cells. And the intestinal barrier and permeability were evaluated by the expression of claudin‐1 and occludin. It was found that the expression of occludin and claudin‐1 in MOL group was significantly higher than that in the L group, suggesting that MOL intervention can improve intestinal barrier function. Next, we explored the changes of intestinal microbes through the bacterial 16SrRNA in the V3–V4 hypervariable region. The gut microbiota has emerged as an internal environment factor closely related to human health. The occurrence and development of polycystic ovary synthesis are closely related to the intestinal flora (Zhao et al., 2020). Besides being associated with changes in serum hormone of PCOS, intestinal flora is also related to the occurrence of inflammation. The chronic low‐grade inflammation is a key contributor to IR in PCOS (He & Li, 2020; Wang, Sha, et al., 2020; Zhu et al., 2020). Furthermore, we analyzed the intestinal microbiota in fecal samples, the correlation between biochemical indices and gut microbiome showed that *Fusobacteria*, *Fusobacterium*, and *Prevotella* exhibited a positive correlation and *Oscillospira* exhibited a negative correlation with the levels of LH, T, LH/FSH, insulin, and HOMAR‐IR. Meanwhile, the relative abundance of *Fusobacterium*, *Prevotella* was markedly increased in the L group than in the C group. Study showed that *Fusobacterium nucleatum* activates TLR4 signaling to MYD88, leading to activation of the nuclear factor NFκB (Yang et al., 2017). Simultaneously, the increase of *Campylobacter* and *Fusobacterium* species can be able to induce inflammation or a variety of opportunistic pathogens of metabolic disorders (Ca Stellarin et al., 2012). Additionally, enrichment of the *Prevotella* has been linked to enhanced IL‐17‐producing cells in colonic mucosa (Iradj et al., 2011). However, the relative abundance of *Fusobacterium*, *Prevotella* was decreased markedly, while the relative abundance *Blautia*, *Oscillospira*, *Adlercreutzia*, and *Parabacteroides* were increased after supplementation MOL. As reported in the literature, Blautia could produce butyric acid and some other short‐chained fatty acids (SCFAs). SCFAs, such as butyrate and acetate, have many benefits and play an important role in intestine function: enhancing the intestinal barrier, improving intestinal microbiota, regulating immune system and promoting gastrointestinal motility, which may have good benefits in reducing the risk of metabolic syndrome (Lin et al., 2021). *Oscillospira*, belonging to the *Clostridial cluster IV*, is an enigmatic bacterial genus possessing potential importance for human health such as negative association with inflammatory diseases and body mass index, Oscillospira also are anaerobes, capable of fermenting complex plant polysaccharides and producing bioactive SCFA with anti‐inflammatory capacity (Konikoff & Gophna, 2016). Meanwhile, *Parabacteroides* is an anti‐inflammatory type of bacteria that produces SCFAs (Liu, Du, et al., 2019), which can offset the slight inflammation caused by the imbalance of bacterial flora. The current result shows that the expression of TLR4 decreased may also be associated with the changed relative abundance of multiple intestinal flora. In this study, we identified that microbial metabolite, and its analog, increases overall gut health by enhancing barrier function in addition to their anti‐inflammatory activities. Therefore, we speculated that MOL upregulated the expression of *Blautia*, *Oscillospira*, *Adlercreutzia*, and *Parabacteroides* and downregulated the expression of *Fusobacteria*, *Fusobacterium*, and *Prevotella*, which in turn downregulated the expression of TLR4 for reducing inflammation to improve the PCOS.

## 5 CONCLUSIONS

In conclusion, the results of this study indicate that MOL supplementation markedly decreased the body weight and restored the estrus cycle, significantly increased the expression of Sirt1, FoxO1, PGC‐1α, IGF1, and modulated the sex hormone level and improved insulin resistance, which may be associated with *Moringa oleifera* leaf supplementation relieves oxidative stress via the Sirt1/FoxO1/ PGC‐1α signaling pathway. Moreover, we have demonstrated that consumption of MOL supplementation results in the alteration of gut microbiota composition and function that was related to the decreased expression of TLR4 and anti‐inflammation. Our results indicate that MOL is potentially a supplementary medication for the management of PCOS. Although much remains to be understood about the comprehensive molecular mechanisms involved in MOL supplementation of PCOS, MOL may be a beneficial direction for PCOS patients.

## AUTHOR CONTRIBUTIONS

**YanXiang Wu:** Methodology (equal); writing – original draft (equal). **XiuYan Yang:** Writing – original draft (equal). **YuanYuan Hu:** Writing – original draft (equal). **XueHong Hu:** Writing – review and editing (equal). **YueLin Zhang:** Writing – review and editing (equal). **Tian An:** Validation (equal). **BoHan Lv:** Validation (equal). **SiYu Tao:** Validation (equal). **Qing Liu:** Conceptualization (equal). **GuangJian Jiang:** Conceptualization (equal); resources (equal).

## FUNDING INFORMATION

This work was supported by Grants from the Basic research projects in Beijing‐Tianjin‐Hebei (19JCZDJC65400(Z)), Horizontal subject of Study on the efficacy and mechanism of hypoglycemic food.

## CONFLICT OF INTEREST STATEMENT

The authors declare no conflicts of interest concerning the authorship and/or publication of this paper.

## ETHICS STATEMENT

The study protocol was approved by the Animal Care and Management Committee of the BUCM. All manipulations were at the request of the guidelines of the Animal Care Committee.

## CONSENT FOR PUBLICATION

Participants agreed to the publication of results.

## Supporting information


Table S1

## Data Availability

The datasets used and analyzed during the current study are available from the corresponding author upon reasonable request.
