# Supplementary material for: Moringa oleifera leaf supplementation relieves oxidative stress and regulates intestinal flora to ameliorate polycystic ovary syndrome in letrozole‐induced rats
Source: Food Sci Nutr. 2023 Jun 6;11(9):5137–56. doi: 10.1002/fsn3.3473 (PMC10494614; doi:10.1002/fsn3.3473)
Supplement: Supplementary file 1 — Table S1 [file FSN3-11-5137-s001.docx]

**Supplementary Table 1.** Measurement of nutrients in dried moringa oleifera leaf.

| **Nutrients** | **Data/100g** | **Testing Method** |
| --- | --- | --- |
| Phosphorus | 900 mg/100g | NY/T 1653-2008 |
| Potassium | 2160 mg/100g | NY/T 1653-2008 |
| Calcium | 2430 mg/100g | NY/T 1653-2008 |
| Magnesium | 313 mg/100g | NY/T 1653-2008 |
| Ferrum | 18.4 mg/100g | NY/T 1653-2008 |
| Zinc | 3.18 mg/kg | NY/T 1653-2008 |
| Cuprum | 0.04 mg/kg | NY/T 1653-2008 |
| Manganese | 5.43 mg/kg | NY/T 1653-2008 |
| Natrium | 9.03 mg/kg | NY/T 1653-2008 |
| Selenium | 0.0107 mg/kg | GB 5009.93-2010 |
| Protein | 24.2 g/100g | GB 5009.5-2010 |
| Crude fat | 8.08 g/100g | GB/T 5009.6-2008 |
| Crude fiber | 6.68 g/100g | GB/T 5009.10-2003 |
| Vitamin C | 110 mg/100g | GB/T 6195-1986 |
| Moisture | 1.2 g/100g | GB 5009.3-2010 |
| Ash content | 6.6 g/100g | GB 5009.4-2010 |
| Total sugar | 14.36 g/100g | GB/T 5009.7-2008 |
| Total starch | 4.56 g/100g | GB/T 5009.9-2008 |
| Total brass | 0.84g/100g | NY/T 1295-2007 |

Notes: Measured by The Ministry of agriculture of agricultural products quality supervision and Testing Center. Sent for inspection by Shandong Grainwood Bio-Technology Company Limited.
